# SMITE: an R/Bioconductor package that identifies network modules by integrating genomic and epigenomic information

**DOI:** 10.1186/s12859-017-1477-3

**Published:** 2017-01-18

**Authors:** N. Ari Wijetunga, Andrew D. Johnston, Ryo Maekawa, Fabien Delahaye, Netha Ulahannan, Kami Kim, John M. Greally

**Affiliations:** 1 Department of Genetics, Albert Einstein College of Medicine, 1301 Morris Park Avenue, Bronx, NY 10461 USA; 2 Division of Obstetrics and Gynecology, Yamaguchi University, 677-1 Yoshida, Yamaguchi Prefecture, 753-8511 Japan; 3 Department of Obstetrics, Gynecology and Women’s Health, Albert Einstein College of Medicine, 1301 Morris Park Avenue, Bronx, NY 10461 USA; 4 Department of Microbiology and Immunology, Albert Einstein College of Medicine, 1301 Morris Park Avenue, Bronx, NY 10461 USA; 5 Department of Pathology, Albert Einstein College of Medicine, 1301 Morris Park Avenue, Bronx, NY 10461 USA; 6 Department of Medicine, Albert Einstein College of Medicine, 1301 Morris Park Avenue, Bronx, NY 10461 USA

**Keywords:** Epigenetic, Gene expression, Modules, Interaction network, Genomic, Bioinformatics

## Abstract

**Background:**

The molecular assays that test gene expression, transcriptional, and epigenetic regulation are increasingly diverse and numerous. The information generated by each type of assay individually gives an insight into the state of the cells tested. What should be possible is to add the information derived from separate, complementary assays to gain higher-confidence insights into cellular states. At present, the analysis of multi-dimensional, massive genome-wide data requires an initial pruning step to create manageable subsets of observations that are then used for integration, which decreases the sizes of the intersecting data sets and the potential for biological insights. Our Significance-based Modules Integrating the Transcriptome and Epigenome (SMITE) approach was developed to integrate transcriptional and epigenetic regulatory data without a loss of resolution.

**Results:**

SMITE combines *p*-values by accounting for the correlation between non-independent values within data sets, allowing genes and gene modules in an interaction network to be assigned significance values. The contribution of each type of genomic data can be weighted, permitting integration of individually under-powered data sets, increasing the overall ability to detect effects within modules of genes. We apply SMITE to a complex genomic data set including the epigenomic and transcriptomic effects of *Toxoplasma gondii* infection on human host cells and demonstrate that SMITE is able to identify novel subnetworks of dysregulated genes. Additionally, we show that SMITE outperforms Functional Epigenetic Modules (FEM), the current paradigm of using the spin-glass algorithm to integrate gene expression and epigenetic data.

**Conclusions:**

SMITE represents a flexible, scalable tool that allows integration of transcriptional and epigenetic regulatory data from genome-wide assays to boost confidence in finding gene modules reflecting altered cellular states.

**Electronic supplementary material:**

The online version of this article (doi:10.1186/s12859-017-1477-3) contains supplementary material, which is available to authorized users.

## Background

In genomics research, the dimensionality of assayed data has increased far beyond the pace of analytical tool development, with data sets likely to continue to increase in size and complexity [1, 2]. We appreciate that gene expression is regulated through a number of interacting mechanisms that include epigenetic processes such as DNA methylation. DNA methylation can also reflect the local binding of transcription factors [3], which are capable of influencing local chromatin structure [4] and post-translational modifications of histones [5]. Furthermore, transcription can induce DNA methylation [6], and DNA methylation can itself influence transcription factor binding [7–11]. While these observations indicate complex interactions between regulators of genomic organization, they also suggest that multiple types of events observed at the same locus increase confidence that regulatory activity is genuinely occurring at that locus. Current methods to explore multiple coincident processes using integrated analysis introduce bias by pruning data sets, either by focusing only on a subset of loci with the most significant effects, or requiring pairwise comparisons of data sets with progressively smaller intersections. Furthermore integrative methods, like Functional Epigenetic Modules (FEM) [12], score genes within a network and identify subnetworks, referred to as modules, but they lack an implemented method to define further functional interpretability an essential outcome of genomics experiment [13]. Therefore, there is a need for a flexible method integrating genomic assay data into a single score that can be used to identify functionally important pathways for further study.

Here we describe an intuitive gene scoring system that combines transcriptional and epigenetic regulatory data sets, an approach we call Significance-based Modules Integrating the Transcriptome and Epigenome (SMITE). The novelty of SMITE lies in the use of mathematic principles and sampling techniques to simplify multiple complex genome-level signals into a single set of interpretable results. We use SMITE to identify novel gene modules in a large, high dimension epigenetic and transcriptomic data set, and we show that SMITE offers improved detection, characterization, and visualization of functional modules within a gene network compared to existing methods. Overall, SMITE provides a useful and intuitive answer to the most important question in integrative genomics: what we can learn from integrating multiple sources of high-resolution information instead of considering each source separately?

## Implementation

### *Toxoplasma gondii (T. gondii)* human foreskin fibroblast data set

To benchmark SMITE and demonstrate implemented features, we obtained a large multifaceted genomics data set from a controlled experiment studying the transcriptional regulatory effects on human foreskin fibroblasts (HFF) following infection by *T. gondii*. Further description of the experimental methods used to produce the data set and results are available in Additional file 1, including alignment to a combined human/*Toxoplasma* genome assembly (Additional file 1: Figure S1).

### Required inputs to SMITE

**Fig. 1:**
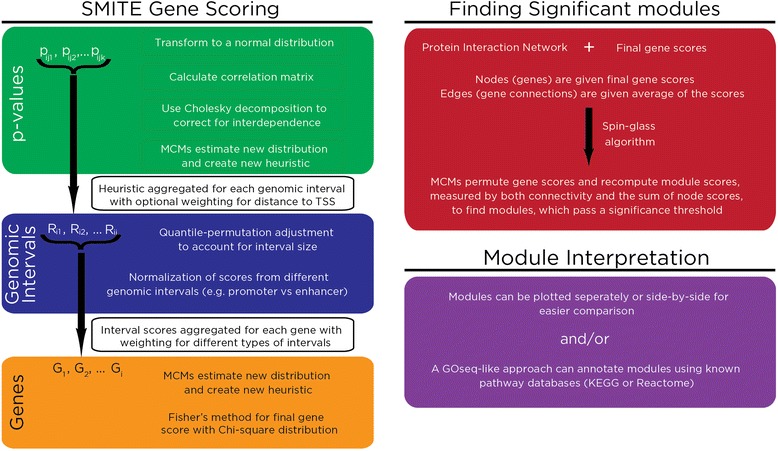
Summary of SMITE. The flowchart details the pipeline through which SMITE takes *p*-values, associates them with genomic intervals, and scores genes. The steps and input required to discover significant modules are shown as well as the downstream functions that SMITE provides for module interpretation

SMITE provides a pipeline that results in annotated functional modules (Fig. 1). It requires the following inputs: 1) a gene annotation bed file, 2) an interaction network, and 3) data sets of effects and statistical test significance from at least one gene expression and/or epigenomic profile(s). In addition, users can include an unlimited number of previously identified genomic intervals of interest (e.g. Chromatin Immunoprecipitation *(*ChIP)-seq peaks, enhancers, Additional file 1: Table S1). Notably, the software relies on *p*-values without specifying the source statistical test, so it is necessary for users to ensure appropriate sample sizes, data quality, and statistical testing of the original experiments.

### Motivation for SMITE

In functional genomics experiments, after performing genomic assays on two or more groups, one generally uses a statistical test to estimate an effect for up to millions of genomic loci (e.g. genome-wide DNA methylation analysis). These estimates are then compared to their standard errors to derive test statistics, *T*, and *p*-values, *p*, where *p* is defined as the probability that *T* is greater than a threshold from a statistical distribution, t, such that *P(T ≥ t) = p*. These test statistics and corresponding *p*-values are used to reject a null hypothesis (i.e. no difference between study groups). While a *p*-value does not represent the probability that a hypothesis is true, in practice, each *p*-value does correspond to a researcher’s relative prioritization of a gene or genomic region within a ranked list [14]. An observed *p*, which increases in significance as it approaches zero, is proportional, ∝, to a new heuristic that is maximized as *1-p* approaches 1, and this heuristic is the probability, *P*, that a gene or genomic region is prioritized by a researcher for further analysis:

1 $$ P\left(T<t\right)=1-p\propto P\kern0.5em \left(\mathrm{Gene}\ \mathrm{or}\ \mathrm{genomic}\ \mathrm{region}\ \mathrm{is}\ \mathrm{prioritized}\right) $$

Therefore, in application, *p*-values are generally reinterpreted beyond their intended purpose, and in this capacity they contribute to new heuristics that are used as the primary criteria for prioritization. While the functional interpretation of significant hypothesis tests from gene expression experiments is straightforward (e.g. genes are significantly upregulated or significantly downregulated), to understand specific functional genomic contexts we must interpret multiple *p*-values as contributing evidence. For example, DNA modifications like DNA methylation and DNA hydroxymethylation are typically measured at the single base pair level, whereas functional genomic contexts are represented by genomic intervals that vary in size, like gene promoters. This necessitates a method of combining multiple *p*-values overlapping the same genomic interval, while also accounting for their likely interdependence. Therefore, these genomic intervals can contribute to a single heuristic that can be used to score their associated genes. Since the relationships between genomic intervals and their associated genes are complex, a flexible approach is needed to allow user input for optimal weighting of genomic contexts depending on a particular experiment.

There are several *p*-value combination methods used in meta-analyses. Because these methods assume independence of experiments, SMITE includes a preprocessing step using Monte Carlo methods (MCMs) to account for non-zero correlations when combining dependent *p*-values [15]. This novel approach implementing MCMs assesses the average strength of the correlations and determine a new distribution of combined *p*-values. Subsequently, *p*-values are recursively combined until every node (gene) in a specific interaction network is associated with a single score that in turn reflects a researcher’s intuitive belief that the node has sufficient evidence to be prioritized for further analysis.

### Combining *p*-values in SMITE

Given *K* experiments with *K* hypotheses, *H*_*i…K*_, test statistics and corresponding *p*-values, *p*_*1…k*_, are calculated so that each *p*-value reflects the probability of observing a particular test statistic or more extreme values; the *p*-value itself is, however, a random variable that follows a uniform distribution, *U*(0,1) [16]. *P*-value combination methods attempt to characterize the joint distributions of two or more of these random variables. If the *p*-values are not independent from one another, then there is a covariance/correlation matrix that needs to be incorporated into the analysis in order to maintain statistical validity. Rather than focus on the statistical distributions of combined *p*-values, which can be complex, difficult to calculate, and risks over-interpreting *p*-values, SMITE uses MCMs, like bootstrapping, to sample randomly a particular set of values from an unknown distribution and to estimate the characteristics of the new combined distribution. SMITE employs these sampling methods before combining large correlated *p*-value data sets. SMITE offers several methods for combining *p*-values including Stouffer’s Z-score method [17] (the default procedure), Sidak’s adjustment [18], Fisher’s method [19], and binomial testing. More detail about the available methods is provided in Additional file 1.

In the idealized scenario, the application of *p*-value combination methods is trivial because of the independence of each epigenetic signal; however, modifications like DNA methylation are thought to be highly correlated over short distances [20], with methods like BumpHunting exploiting this local correlation to define differentially methylated regions [21]. For this reason, SMITE estimates the average correlation between the dependent *p*-values as a function of distance. For each gene *G*_*i*_ for *i* in 1,2…*I*, we first find the *J* genomic intervals *R*_*ij*_ for *j* in 1,2…*J* related to *G*_*i*_ (e.g. a specific gene’s promoter and body). Then, we determine the *N* overlapping *p*-values, *p*_*ijk*_ for *k* in 1…*N*_*ij*_, for each genomic interval. Next, we convert the *p*-values to a standard normal distribution with the transformation *Z*_*ijk*_ = *Φ* ^*−1*^ *(1 − p*_*ijk*_ */2)*, where Φ is the standard normal cumulative distribution function (CDF). Rather than incorrectly assuming that the *p*-values are independent, we chose to use a non-parametric MCM approach to estimate correlation coefficients for modifications that overlap the same interval, *R*_*ij*_.

**Fig. 2:**
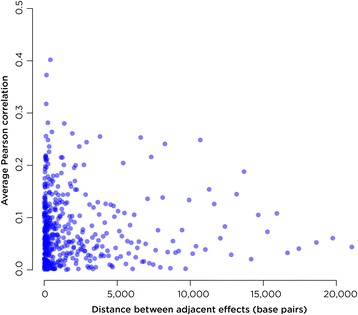
Monte Carlo simulation of correlation matrix for DNA methylation. The average Pearson correlations as a function of distance separating adjacent effects for DNA methylation in the *T. gondii* HFF data set. As expected, there is general decrease in the correlation of DNA methylation values as the distance between assayed sites increases

We estimate a correlation matrix using the physical distance between loci associated with *p*-values, and thus, we control for a background level of spatial correlation. To estimate this matrix, we find for each significant *p*-value within a type of interval *R.*_*j*_ the distances to the closest upstream and downstream *p*-value. As HELP-tagging [22] and Illumina HumanMethylation450 BeadChip array [23] data have ~2 million data points and ~450,000 probes, respectively, these distances were binned in 500 bins, resulting in as little as single basepair bins for the smallest distances, where we expect the largest correlations. We randomly sampled within bins with replacement and found the Pearson correlation between the transformed *p*-values. This process was repeated 500 times and the average correlation was associated with the bin. The results from a correlation matrix using DNA methylation from the *T. gondii* HFF data set indicate, as expected, that the estimated correlation is generally higher between *p*-values close to one another, and that it tends to decrease with distance (Fig. 2). Even when these correlations are small, it is inappropriate to ignore them completely, and this calculation is necessary to account for the background interdependence of effects.

Having determined a correlation matrix, ${\varSigma}_{ij}$, that is symmetric, positive, and definite, we can determine an upper triangular matrix with positive diagonal entries using the Cholesky decomposition, *C*_*ij*_, so that ${\varSigma}_{ij}$ *=C*_*ij*_^*T*^ *C*_*ij*_, and this decomposition can be used to adjust the previously transformed *p*-values Z_ijk_, where *Z*_*ijk*_ = *Φ* ^*−1*^ *(1 − p*_*ijk*_ */2)*, such that [16]:

2 $$ {C_{ij}}^{-1}{\varPhi}^{-1}\left(1-\frac{p_{ijk}}{2}\right)={Z}_{ijk}^{*} $$

Through this method the correlated *Z*_*m*_ and *Z*_*n*_ for *m ≠ n* are now approximately independent and can be combined as independent experiments. The Cholesky decomposition is discussed in greater detail in Additional file 1. Additionally, SMITE employs MCMs to estimate the distribution of the combined statistics so that the new *p*-values can be thought of as completely new heuristics indicating confidence in a particular *p*-value, ${Z}_{ijk}^{*}$.

**Fig. 3:**
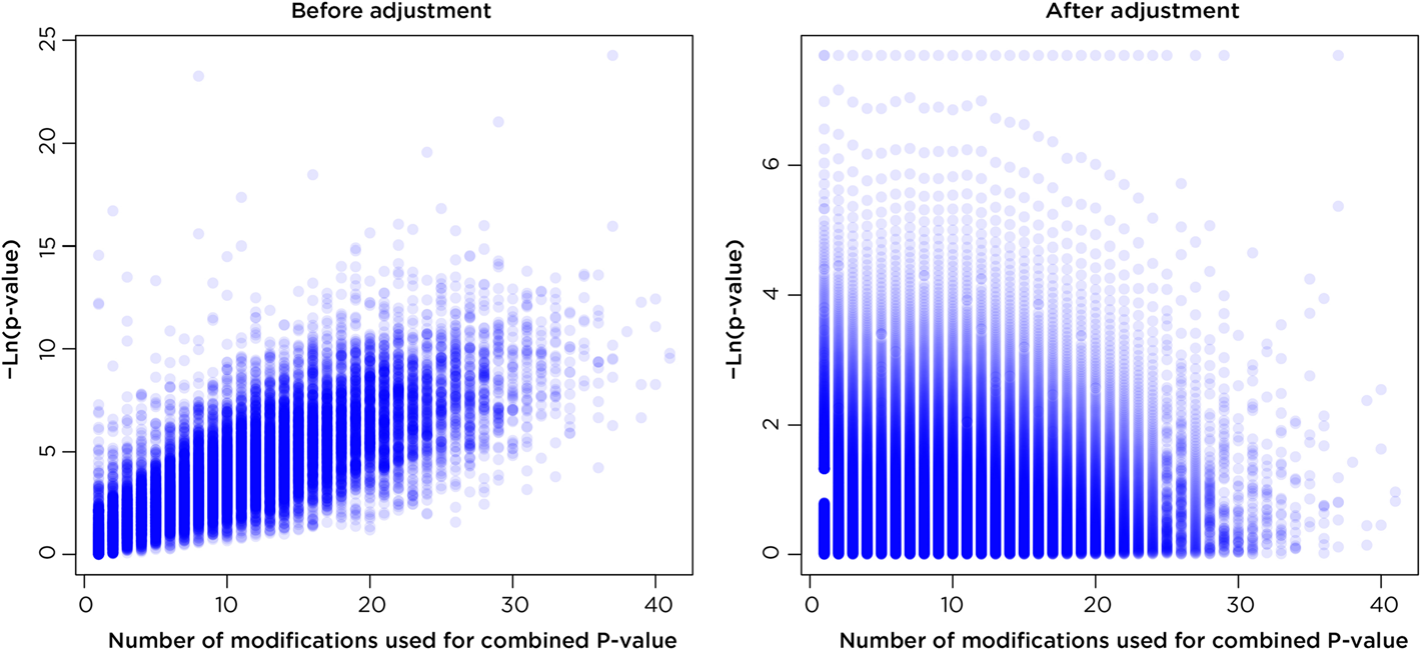
The effect of adjustment by the total number of combined *P*-values. In this example taken from the *T. gondii* HFF data set, the negative natural log of the significance of the combined *p*-value is plotted against the number of *p*-values that were combined for each value. The increased trend is visible before adjustment (*left*) and is no longer present after adjustment (*right*)

An aggregated score, *R*_*ij*_, is calculated using the weighted Stouffer’s method:

3 $$ {R}_{ij}=\frac{{\displaystyle {\sum}_{k=1}^N}{w}_{ijk}{Z}_{ijk}^{*}}{\sqrt{{\displaystyle {\sum}_{i=1}^k}{w}_{ijk}^2}}\sim N\left(0,1\right) $$

where *w*_*ijk*_ represents optional weights such as distance from the gene transcription start site (TSS) [24]. An analysis where no weights *w*_*ijk*_ are used is shown in Additional file 1: Figure S2 where an R^2^ = 0.99 between final scores with and without weighting and nearly identical final modules and annotations in Additional file 2: Tables S14–S15, indicate that SMITE is robust for choices of *w*_*ijk*_ *.* In a high-resolution epigenomic assay like HELP-tagging, it is possible to have as many as ~2000 data points (*p*-values) associated with a large region like a gene body. Because aggregated scores increase as the number of *p*-values within a genomic interval increases, SMITE implements a quantile-permutation adjustment, whereby a specific *R*_*ij*_ is compared to 100 distributions of randomly sampled *R’*_*ij*_ scores from the same *N*_*ij*_ quantile. We estimate p*_ij_, the proportion of sampled *R’*_*ij*_ scores at or more extreme than the observed *R*_*ij*_ and $\overline{p}$ *_ij_, the average of the proportions from random samples. Finally, we consider *R*_*ij*_ = Φ ^*−1*^ *(1−* $\overline{p}$ *_ij_ *)* with an effect direction (e.g. less or more DNA methylation) derived from the *p*-value effect sizes. The improvement after controlling for the number of combined *p*-values on the combined significance can be seen before and after adjustment (Fig. 3).

### Normalization of aggregated *p*-value-derived scores

**Fig. 4:**
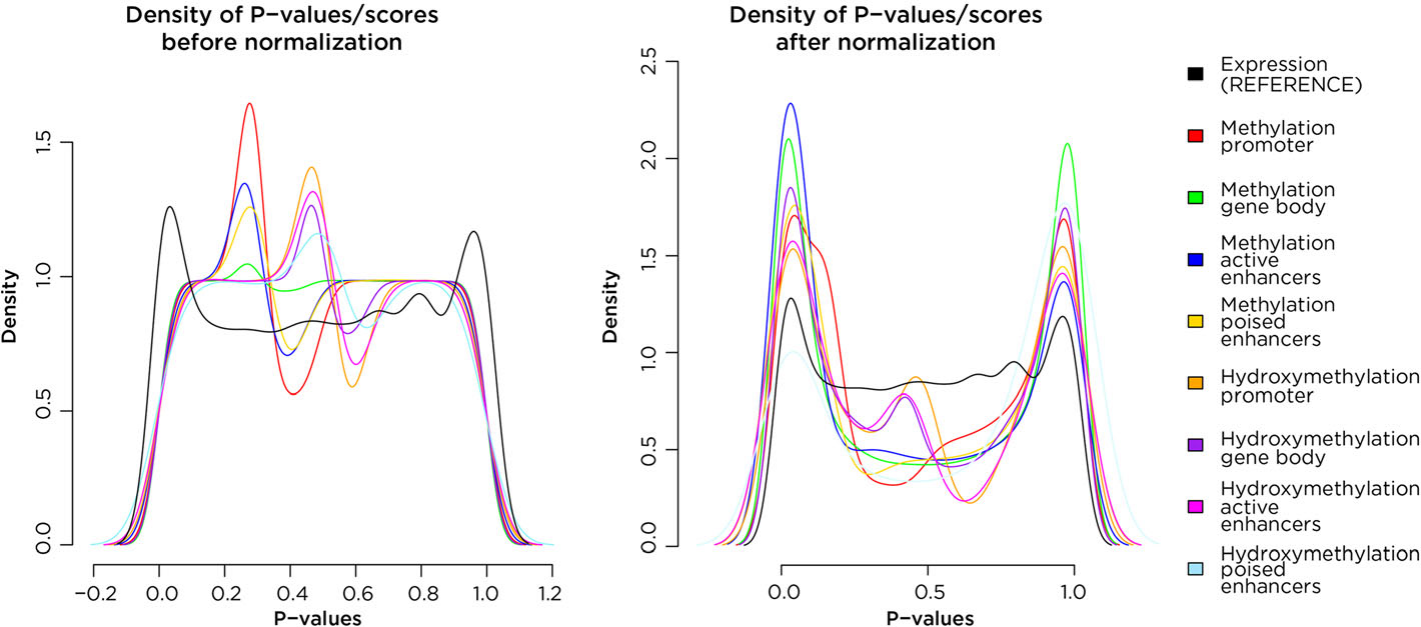
Normalization of combined *p*-value scores. The densities of the scores/*p*-values for the *T. gondii* HFF data set are plotted using the SMITE functions to compare each of the annotated contexts to determine if normalization is necessary (*left*). After normalizing the values by logit transformation, rescaling, and back-transformation, the densities of the normalized *p*-values are shown (*right*)

We found that despite each component score *R*_*ij*_ being normalized for the number of combined *p*-values, a slight difference in the distribution of one component can drive downstream scores and bias module detection. To resolve this potential limitation, we implement a normalization step that results in more comparable component scores, R_ij_, for all genes (i in 1,2,…I). There are two methods available for normalizing scores depending on the distribution of the combined *p*-values and both represent monotonic transformations preserving the order of the scores. The first available method is a logit transform of the *p*-values, followed by rescaling to a common scale and then recovering the adjusted *p*-value. This method has minimal effects on the actual data, but it successfully improves the overall distribution and comparability of the different types of data (Fig. 4). The second available method is a variation on Box-cox transformations where an iterative process identifies an optimal power transformation of the data.

**Fig. 5:**
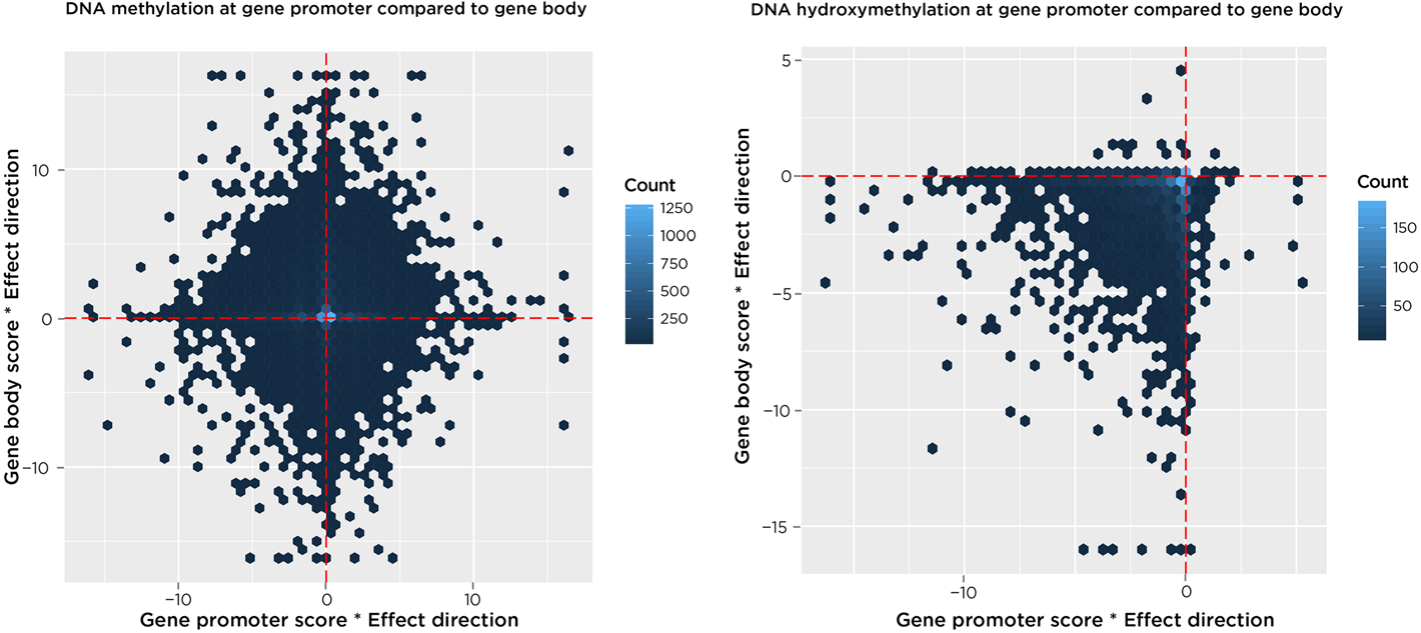
Epigenetic modifications at promoters compared with gene bodies. Using the SMITE functions, we show a comparison of the component scores (the –ln (*p*-value) version of the Score) and the effect direction for gene promoters and gene bodies in the *T. gondii* HFF data set. For DNA methylation (*left*), there is not a large relationship between scores and directions of scores between promoters and bodies, whereas for DNA hydroxymethylation (*right*) there is a concordance of loss of hydroxymethylation in promoters and gene bodies

The comparison of R_ij_ (e.g. the gene expression scores compared to the gene promoter DNA methylation scores for the same gene) can provide useful information about the overall observed trends. Here, we show a comparison of the gene promoter scores with gene body scores for DNA methylation and DNA hydroxymethylation in the *T. gondii* HFF data set, and we can see that hypo-hydroxymethylated gene bodies are associated with hypo-hydroxymethylated promoters (Fig. 5).

### Final score derivation for downstream analysis

Finally, we derive a single score for each gene, *G*_*i*_, using the Stouffer method again, with optional weights *w.*_*j*_ for each *R.*_*j*_ reflecting the researcher’s main analysis goals (e.g. increased weighting for gene expression and DNA methylation at gene promoters), including a directionality coefficient *B.*_*j*_ reflecting a researcher’s a priori understanding about the relationship between each *R.*_*j*_ (e.g. increased DNA methylation at a gene promoter is correlated with decreased gene expression [25]). Because the combined score represents linear combinations of weights and transformed *p*-values, we again use MCMs by bootstrapping to determine a new adjusted *p*-value for each gene, p_i_. Scores for each gene are then calculated using Fisher’s method as *G*_*i*_ *= −*2*ln(p*_*i*_ *)*, which has an approximate Chi-square distribution with 2° of freedom. High scoring genes can be used for other analyses such as Gene Set Enrichment Analysis [26] and network-based approaches.

To explore the impact of weight choice for each *R.*_*j*_ on downstream analysis, we fixed the weight values *w.*_*j*_ for j in 1,2…J, varied the one individual weight *w.*_*m*_, for *m* not in {1,2…J} and for each variation, we extracted the highest scoring genes using a sampling approach with replacement to determine the background score distribution. This analysis allowed us to assess how individual gene’s scores varied with weight choice, and to what extent the overall high scoring geneset was altered in Additional file 1: Figure S3. As expected, we observe that as the relative weighting increases, the effect of each *R.*_*m*_ on the overall identified geneset is greater; however, roughly 50% of the identified genes remain constant, likely depending mostly on other *R.*_*j*_ for m *≠* j for their overall scores. For each *R.*_*m*_, as *w.*_*m*_ increases, a different subset of genes emerges that likely depends on *R.*_*m*_ (i.e. there are associated significant *p*-values). Ultimately, we believe this flexibility in identified genes is a strength of the technique as it allows the researcher to identify a subset of genes that is robust to weight choice, but also allows for overall gene sets that differ depending on *R.*_*j*_ of interest.

### Module identification within SMITE

In SMITE, modules are identified by inputting scores into a spin-glass algorithm as in Epimods [27] or a heinz algorithm [28] as in BioNet [29]. The spin-glass algorithm in network analysis was initially suggested by Reichardt and Bornholdt [30] who sought a method of defining subsets of nodes within a network that were more densely interconnected, suggesting that these represented a joint spin state, or community. They proposed that the relative density of the connections, called modularity, could be compared to modularity under a null distribution to derive significant communities within a larger network. The spin-glass algorithm, which depends on a single parameter [31], has been shown to an effective method for finding modules as long as its parameter is set below 0.6, and in fact, it was shown that fixing this parameter at 0.5 results in an optimal number of genes within a module [27]. Thus, SMITE also uses a 0.5 parameter for running the spin-glass algorithm. Alternatively, the Heinz algorithm uses a linear programming approach called *branch-and-cut* where connections between nodes are converted to two directed edges and trimmed until a single optimal subnetwork is identified. Thus in practice, the Heinz algorithm produces a larger summary subnetwork of genes that typically encompasses the separate modules found using the spin-glass algorithm.

Whereas other subnetwork identification algorithms define significance on the basis of observed subnetwork modularity (i.e. connectivity), SMITE allows modules to have both connectivity significance and an additional associated statistical significance related to the sum of the individual node within a module. Because our scores are derived from *p*-values, we employ Fisher’s method mentioned above to assess the overall module significance, which should follow a Chi-square distribution with *2 k* degrees of freedom, where *k* is the number of genes within a module (see Additional file 1: Supplementary methods). Therefore, this significance can be used to rank and filter modules.

## Results and discussion

### Integrative analysis increases study power

SMITE increases the power of analysis at four levels: (1) by analyzing combined genomic signals from multi-level genomics experiments and avoiding the inflated type I error that characterizes pairwise comparisons of genomic signals; (2) by combining incomplete data sets so that having one missing signal will not eliminate a gene from analysis; (3) by allowing prioritization of the most important signals and genomic contexts (a subjective criterion dependent on research goals) for further downstream analysis; and (4) by implementing methods to analyze groups of genes within networks or pathways together. We have therefore designed SMITE to aid in the interpretation of integrated data that were given rigorous statistical treatment during upstream analysis. In the setting of underpowered, preliminary research, SMITE is better used as an exploratory tool to help target downstream analysis and plan further experiments.

### SMITE identifies novel dysregulated functional modules in *T. gondii*-infected human cells

In Additional file 1: Table S2, we show two sets of criteria that we used to score the *T. gondii* HFF data called reduced (SMITE-R) and full (SMITE-F) models that illustrate how a researcher can use SMITE with varied weighting to identify varied gene modules. The SMITE-R model only includes gene expression and gene promoter DNA methylation; whereas in the SMITE-F model also includes enhancer (active and poised) and gene body DNA methylation and hydroxymethylation. We were primarily interested in transcriptional regulatory alterations at enhancers (histone H3 lysine 4 monomethylation, H3K4me1) and how those relate to functional annotations, so in SMITE-F, enhancer-defining marks were weighted highest, followed by gene expression, gene promoters, and gene bodies. As mentioned previously, we expect that DNA methylation should have a negative correlation with gene expression at gene promoters [32], and a positive correlation with gene expression at gene bodies [33–35]. In contrast, for the purpose of this demonstration, we do not assume any known relationship between DNA methylation at enhancers or for DNA hydroxymethylation at any genomic feature. For both the reduced and full models, we ran the spin-glass and the Heinz algorithms. For the spin-glass algorithm, we requested modules that had at least 8 genes but no more than 100 genes. For the heinz algorithm, we input a subset of high scoring genes identified by randomly sampling the scores to find the background distribution. The R code that we used is shown in Additional file 1: Appendix 1, and the list of genes within the summary network generated by the heinz algorithm is shown in Additional file 2: Table S11.

**Fig. 6:**
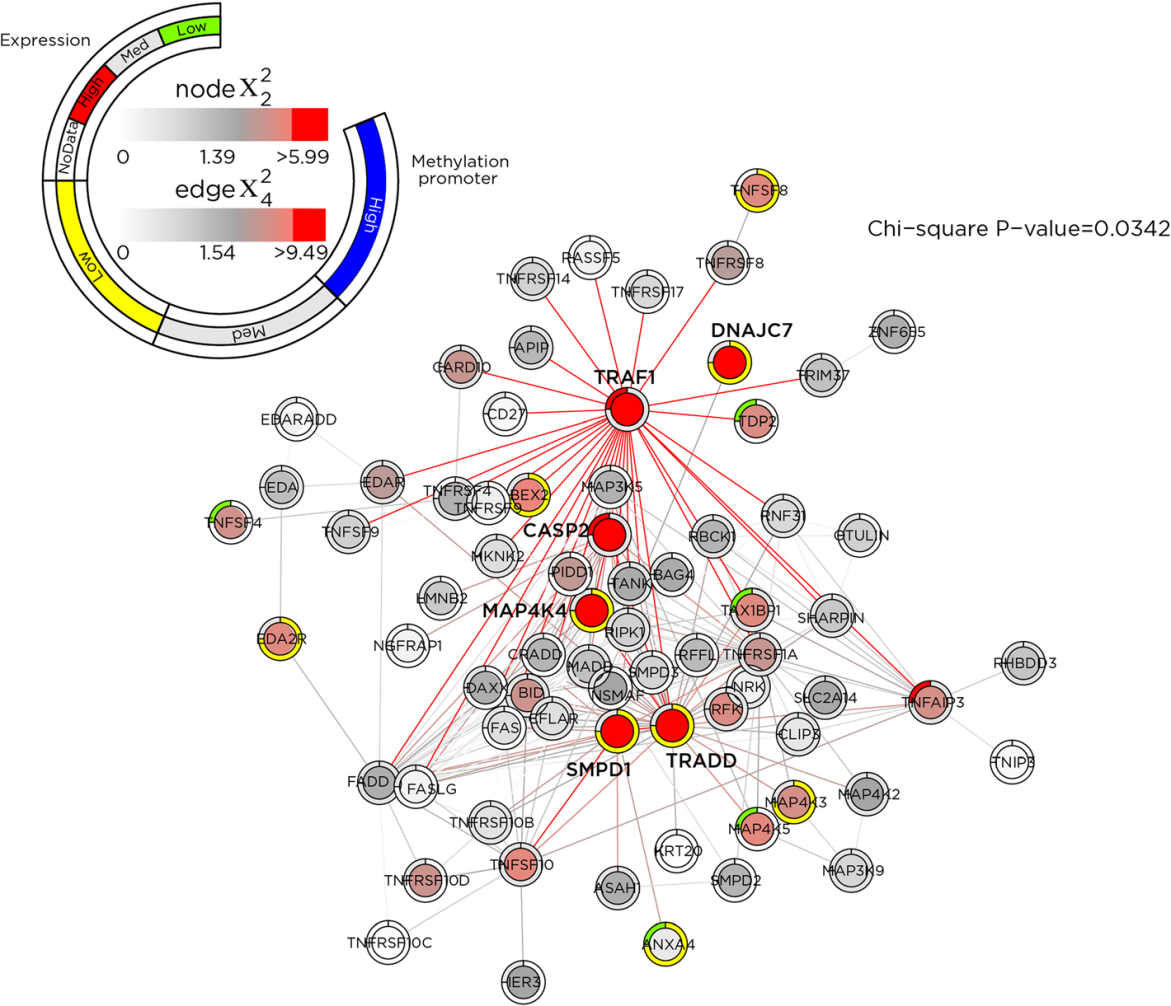
SMITE-identified module implicating cell cycle and MAPK pathways. SMITE allows visualization of the relationship between each component score and the overall node score. This functional module is enriched in human genes that regulate cell cycle by altering cell survival and apoptosis consistent with the known property of *T. gondii* infection of human cells to induce host cell cycle arrest at G2. The module shows *MAPK4* as a highly scoring gene (intense red coloring) centered within the network

**Fig. 7:**
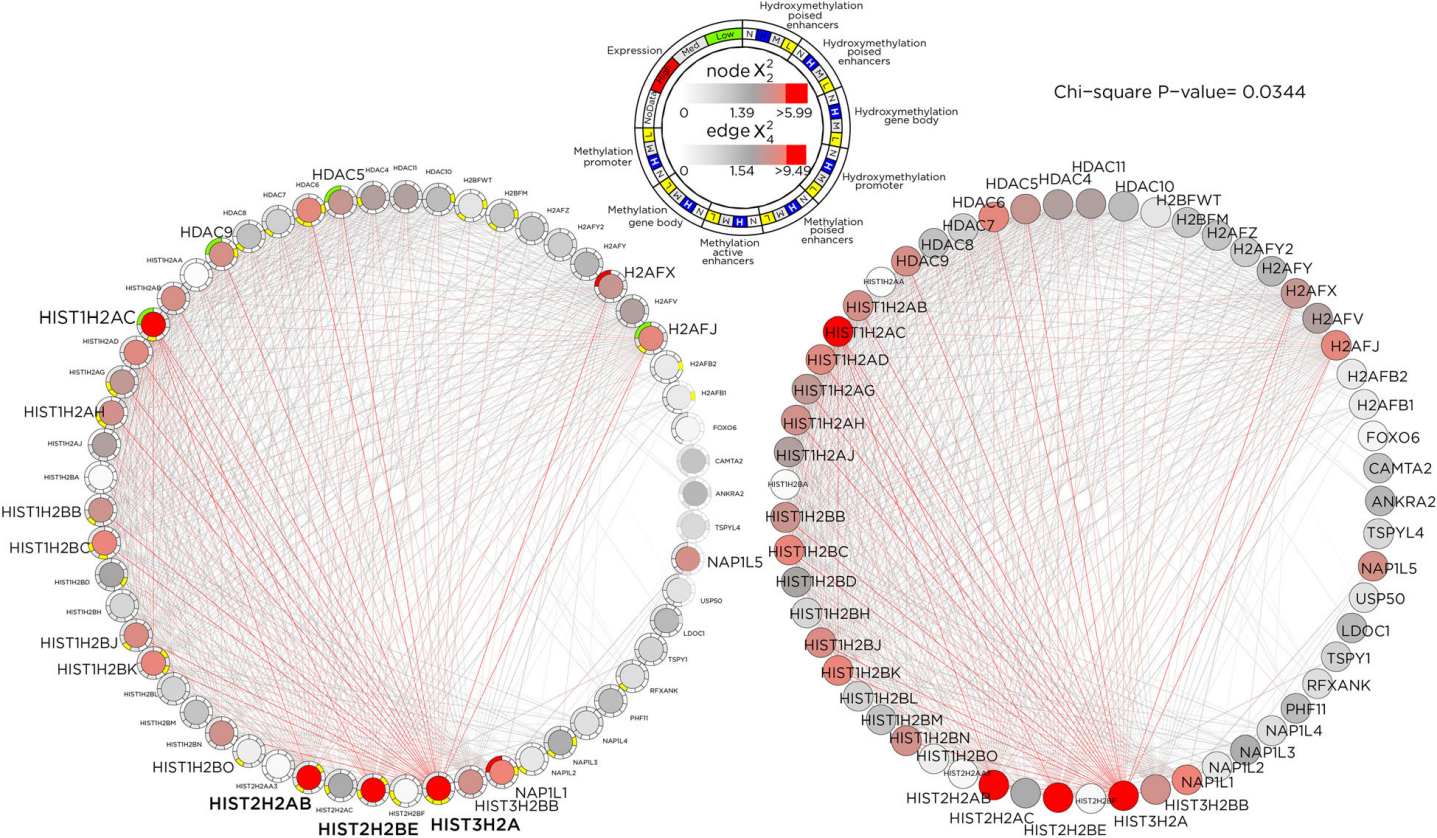
SMITE-identified module implicating chromatin regulation. The module centered around histones and their regulators is plotted in a circular layout in two modes, with (*left*) and without (*right*) component score details. We can see that many of these genes were implicated because of their component scores for gene expression and events occurring at enhancers

The effect of SMITE-R and SMITE-F model choices on the overall scores is shown in Additional file 1: Figure S4. Through the spin-glass algorithm, both SMITE-R and SMITE-F identified 13 modules representing 528 and 510 genes, respectively (Additional file 2: Tables S5–S6), with an overlap of only 94 genes. Notably, four and two of the 13 modules for SMITE-R and SMITE-F, respectively, showed enrichment for infection-related and inflammation-related annotations, as would be expected for infection of a host cell by an intracellular pathogen. In addition, we find that generally metabolism-related modules are dysregulated in five and four of the 13 modules for SMITE-R and SMITE-F, respectively, suggesting that host cell metabolism may be altered after infection. For SMITE-R, two modules enriched for cell cycle and apoptosis related effects confirming prior observations regarding *T. gondii* infection in host cells [36–39]. In Fig. 6 we show one cell cycle related functional module identified by SMITE-R that also indicates altered MAPK signaling, a previously implicated feature in toxoplasmosis of mice [40, 41] and humans [42]. While it has been demonstrated that *T. gondii* infection of human cells induces host cell cycle arrest at G2 [37, 38], the identified module indicates that *T. gondii* may accomplish this through combined epigenetic dysregulation at promoters and transcriptomic dysregulation. In SMITE-F, three modules strongly implicate chromatin remodeling, epigenetic regulation of gene expression, and detection of pathogen DNA in the cytosol, and in Fig. 7, we show an identified module with multiple epigenetic events at genes’ active and poised enhancers. Results from the Heinz algorithm are concordant in showing many cell cycle related pathways for the reduced model and additional altered cell signaling pathways in the full model (Additional file 1: Figure S5, Additional file 2: Table S12–S13). Therefore, SMITE analysis suggests that *T. gondii* infection remodels the epigenome of the infected host and alters host gene expression, impacting host gene networks that regulate metabolism, intracellular signaling, and cell cycle progression, and these findings are part of a manuscript in preparation (Ulahannan et al.,). To ensure robustness of results, we performed the analysis twice more, and despite using random sampling procedures at multiple points within SMITE, we obtained the same modules and module significance each time, indicating that SMITE results are highly reproducible. More detail about each module is given in Additional file 2: Tables S5–S6 and Tables S7–S9.

### SMITE improves integrative genomics methods

We identified FEM and BioNet as computationally efficient methods to identify gene modules, and we designed SMITE to improve the gene scoring functionality of these technologies. While SMITE can serve as a wrapper for module-identifying functions of FEM and BioNet, there are several major shortcomings of these approaches, which we have addressed with SMITE. Although these improvements preclude a direct head-to-head comparison of SMITE to other methods, a discussion of these improvements illustrates the novel aspects of SMITE as compared to state-of-the-art technology.

Both SMITE and BioNet use *p*-values as an input, while FEM usually employs t-statistics that have been averaged over a region near the transcription stat site (TSS). By averaging t-statistics over a region directly adjacent to the TSS, FEM does not preserve the biology of epigenetic processes like DNA methylation that may occur far from the TSS and may not occur equally throughout a region. Though FEM is not limited to T-tests, the algorithm assumes sample normality and uses scaling of the relationship between DNA methylation and expression by the ratio of the t-statistic variances – a technique that is optimal for combining T-tests. Therefore, FEM is only functionally optimal for analyzing T-tests, which is often inappropriate in genomics considering data distributions and the necessary adjustments for confounders such as experimental batch effects [43]. Thus, the *p*-value is a more versatile input because it can be derived from different statistical methods depending on each individual experiment.

FEM can only integrate one epigenetic modification, usually DNA methylation, with gene expression. If a researcher wanted to compare multiple types of epigenetic data with expression and with each other, it would necessitate either pairwise comparisons between each epigenetic dataset and expression, which would hinder the overall study interpretation, or manual selection of a single *p*-value for each gene, which would bias the findings. Though BioNet allows several *p*-values to be associated with a gene so that more than one epigenetic modification could be integrated, it does not have an implemented method to arrive at a single summary statistic or *p*-value for the epigenetic modifications, again requiring manual curating of the input data. To address these major shortcomings, SMITE uses a statistically sophisticated aggregation and normalization algorithm that that allows the user to input *p*-values and multiple genomic intervals, thus allowing simultaneous comparison of many types of data including, but not limited to, DNA methylation, DNA hydroxymethylation, and ChIP-seq peak data.

BioNet does not incorporate the effect direction its scoring method, and FEM incorrectly assumes that the epigenetic modification statistic will always have an inverse relationship with gene expression, which oversimplifies the complexity of gene expression regulation. To address this limitation, SMITE is novel in allowing the user to adjust the directionality of an epigenetic modification’s relationship with gene expression in a genomic context-dependent manner.

In addition, FEM has a very specific input structure that requires rows of the DNA methylation data, expression data, and graph objects to have matching Entrez gene ids. Unfortunately, this may not be straightforward to assemble and will negatively select genes that only have partial data (e.g. having only gene expression or only DNA methylation) or are not part of an interaction network. Functionally, each FEM analysis becomes centered around the nodes that are still available in a specific interaction network instead of centered around high scoring genes regardless of missing data. BioNet employs non-parametric order statistics that ignore missing data. Because SMITE uses a combined *p*-value for each node, it does not specifically require a high scoring node to have complete data. SMITE is also not limited by gene annotation (e.g. Entrez, Refseq) as a consistent set of identifiers is used. Thus, SMITE allows for missing data and flexibility of gene annotation.

Finally, FEM and BioNet rely on ranking genes based off the sum of their DNA methylation and gene expression statistics and a combined *p*-value, respectively. In contrast, SMITE is novel in allowing users to input a prioritization of genomic contexts relative to one another so that the identified functional modules reflect the researcher’s goals or intuition. Therefore, the findings in SMITE are more robust for novel pathway discovery and exploratory analysis.

### Comparison of modules detected using SMITE and FEM

Though SMITE and FEM are not directly comparable, having shown that SMITE can identify functionally important modules within the *T. gondii* HFF data set, we aimed to demonstrate that SMITE-identified modules are not the same as those identified by FEM. Additionally, because the spin-glass algorithm can identify several modules compared to a single module in BioNet, a comparison of the multiple identified modules between SMITE and FEM allows more resolution. To compare SMITE and FEM, we used the criteria defined in the FEM vignette to associate genes with DNA methylation. We calculated t-statistics with four degrees of freedom for gene expression and DNA methylation analysis, and we associated DNA methylation with genes by: 1) taking the average of all effects within 200 bp from a gene transcription start site (TSS), 2) if no effects were found, taking the average of effects over the first exon, and 3) if no effects were found, taking the average over 1500 bp around the TSS. The R code that we used to run FEM is shown in Additional file 1: Appendix 2. The high-scoring genes identified by the three models (SMITE-F, SMITE-R and FEM) are listed in Additional file 2: Table S3. We compare the FEM model with the SMITE-R model, which is directly comparable because it equally weights gene expression and promoter DNA methylation and in opposite directions, and the SMITE-F model, which incorporates additional information regarding gene enhancers.

**Fig. 8:**
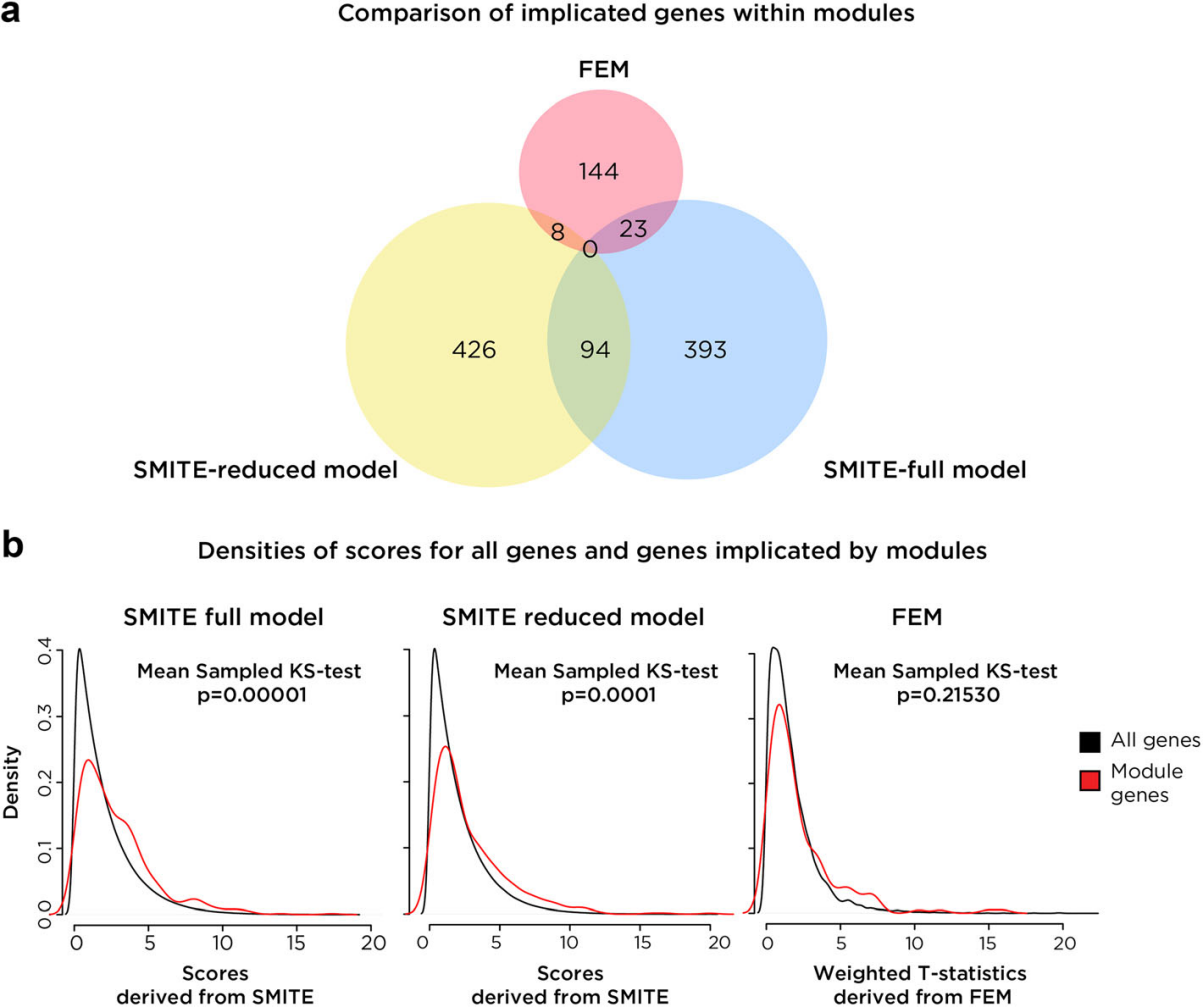
SMITE comparison with FEM. **a** An Euler diagram showing that no genes were found by all three models: FEM, SMITE-R, and SMITE-F. SMITE-F and SMITE-R overlap much more than either do with FEM. **b** A comparison of the densities of all scores compared to genes identified within modules by SMITE-F (*left*), SMITE-R (*middle*), and FEM (*right*), indicating that there is a statistically significant enrichment for high scoring genes using SMITE even when using the reduced model

We used the *DoFEM.bi* function in FEM with the default settings provided in the FEM package vignette. Using FEM we identified 7 modules that have between 8 and 100 genes (Additional file 2: Tables S4 and S7). In summary, FEM implicated 175 genes, only 8 of which overlapped those identified with the reduced SMITE model and 23 of which overlapped those identified by SMITE-F (Fig. 8a). Therefore, since SMITE-F identified modules represent combined gene expression and DNA methylation and DNA hydroxymethylation at enhancers, and the SMITE-R and FEM-identified modules only focus on DNA methylation at gene promoters and expression, the techniques appear to identify largely different modules and genes. Additionally, SMITE-R and FEM models appear to also identify mutually exclusive genes. Though FEM does not have an implemented method to examine further pathway annotations, we annotated it using GoSeq and compared enriched pathways. In Additional file 2: Table S10, it is apparent that all three models enrich for metabolism, signal transduction, and the immune system to some extent; however, while FEM and SMITE-R model enrich for cell cycle regulation, only the SMITE models indicate transcriptional regulatory processes.

We then examined how each technique was able to enrich for high scoring nodes within identified functional modules. In Fig. 8b we compare the density of all scores compared to the density of scores for genes within modules for FEM, SMITE-R and SMITE-F. SMITE-R and SMITE-F have a statistically different distribution (simulated Kolmogorov-Smirnov (KS) test *p* = 0.00001 and *p* = 0.00001, respectively) of enriched genes compared to all scored genes whereas FEM contains the equivalent of a random sampling of scored genes (Kolmogorov-Smirnov test *p* = 0.2153). The derivation of the KS-test significance for these tests is shown in Additional file 1: Figure S6.

Finally, in Additional file 1: Figure S7 we show the relationship between high scoring genes and the number of *p*-values associated with those genes. Because the FEM input involved averaging *p*-values in discrete regions around the TSS, the highest scoring genes in FEM tend to be biased by having more associated *p*-values when compared to high scoring genes in the full-SMITE model (KS test *p* < 10^−12^).

The limitations of FEM make it impossible to perform a head-to-head comparison with SMITE to identify simulated effects occurring at putative enhancers and incorporating DNA hydroxymethylation. Nevertheless, assuming the existence of true functional modules that represent interconnected genes that are dysregulated by common epigenetic mechanisms within a pathway, SMITE enriches for genes that are high scoring and is, therefore, very sensitive and specific. In contrast, FEM modules will tend to have many low scoring nodes, which may indicate that FEM is not as sensitive, or there may be many more false positives within FEM modules. FEM genes are also biased by having a higher number of associated *p*-values. Therefore, we conclude that the heuristic used to prioritize genes in SMITE employs a robust algorithm that integrates multi-level genomics findings and can identify novel functional modules that are both focused and meaningful.

## Conclusions

Current genomic experiments are underpowered to detect genomic events comprehensively within a network, and a functional module identified by SMITE is implicated by the cumulative evidence of varied input data over all of its members. Modules implicate potentially important network members for which there may be no statistically significant evidence. Thus, SMITE is a discovery platform to integrate multi-level genomic observations that represents a significant improvement over existing integrative genomics approaches. Through SMITE, researchers can increase study power to find a single set of interpretable results integrating epigenomic and transcriptomic data sets.

## Additional files

Additional file 1: Supplementary Methods. **Figure S1** Proportions of RNA-seq reads from *T. gondii*-infected HFFs aligning to a composite hg19/*Toxoplasma* genome. **Figure S2** Comparison of distance weighting effect on gene scores. **Figure S3** Representation of simulations demonstrating the effects on high scoring genes of variation of weightings. **Figure S4** Comparison of gene scores with reduced and full SMITE models. **Figure S5** Examples of modules generated by full and reduced SMITE models. **Figure S6** KS test results comparing SMITE and FEM module genes and a random sampling of 10,000 genes. **Figure S7** Comparison of the performance of the full SMITE model with the FEM model. **Table S1** Criteria for defining genomic contexts in HFFs. **Table S2** Weighting criteria used for SMITE analysis of the *T. gondii* HFF dataset. **Appendix 1** R code for analyzing *T. gondii* HFF dataset with SMITE. **Appendix 2** R code for analyzing *T. gondii* HFF dataset with FEM. Supplementary references (PDF 5642 kb)
Additional file 2: Supplementary Tables. **Table S3** Gene symbol and score of the high scoring genes using three different methods: SMITE full model, SMITE reduced model, and FEM. **Table S4** Modules discovered using FEM and genes composing the modules with their DNA methylation, expression, and overall statistics. **Table S5** Modules discovered using the reduced model of SMITE (SMITE-R) with spin-glass. **Table S6** Modules discovered using the full model of SMITE (SMITE-F) with spin-glass. **Table S7** Pathways associated with the genes composing the modules discovered by FEM. **Table S8** Pathways associated with the genes composing the modules discovered by the reduced model of SMITE (SMITE-R) using spin-glass. **Table S9** Pathways associated with the genes composing the modules discovered by the full model of SMITE (SMITE-F) using spin-glass. **Table S10** Quantifying the number of times pathways were found to be associated the modules discovered by either FEM, the reduced model of SMITE (SMITE-R) using spin-glass, or the full model of SMITE(SMITE-F) using spin-glass. **Table S11** Genes composing the “summary network” found by either the reduced (SMITE-R) or full (SMITE-F) SMITE models using the Heinz algorithm. **Table S12** Pathways associated with the genes composing the “summary network” discovered by the reduced model of SMITE(SMITE-R) using the Heinz algorithm. **Table S13** Pathways associated with the genes composing the “summary network” discovered by the full model of SMITE (SMITE-F) using the Heinz algorithm. **Table S14** Genes composing the “modules” found using no weights instead of weighting by distance. **Table S15** Pathways associated with the genes in the modules identified without using distance weighting. (XLSX 269 kb)

## Abbreviations

ChIP: Chromatin immunoprecipitation
FEM: Functional Epigenetic Modules
HFF: Human foreskin fibroblasts
MCM: Monte Carlo method
SMITE: Significance-based Modules Integrating the Transcriptome and Epigenome
TSS: Transcription start site
