# Supplementary material for: SMITE: an R/Bioconductor package that identifies network modules by integrating genomic and epigenomic information
Source: BMC Bioinformatics. 2017 Jan 18;18:41. doi: 10.1186/s12859-017-1477-3 (PMC5242055; doi:10.1186/s12859-017-1477-3)
Supplement: Additional file 1: — Supplementary Methods. Figure S1 Proportions of RNA-seq reads from T. gondii-infected HFFs aligning to a composite hg19/Toxoplasma genome. Figure S2 Comparison of distance weighting effect on gene scores. Figure S3 Representation of simulations demonstrating the effects on high scoring genes of variation of weightings. Figure S4 Comparison of gene scores with reduced and full SMITE models. Figure S5 Examples of modules generated by full and reduced SMITE models. Figure S6 KS test results comparing SMITE and FEM module genes and a random sampling of 10,000 genes. Figure S7 Comparison of the performance of the full SMITE model with the FEM model. Table S1 Criteria for defining genomic contexts in HFFs. Table S2 Weighting criteria used for SMITE analysis of the T. gondii HFF dataset. Appendix 1 R code for analyzing T. gondii HFF dataset with SMITE. Appendix 2 R code for analyzing T. gondii HFF dataset with FEM. Supplementary references (PDF 5642 kb) [file 12859_2017_1477_MOESM1_ESM.pdf]

SUPPLEMENT

SMITE: an R/Bioconductor package that identifies network modules by integrating genomic and epigenomic information.

N. Ari Wijetunga, Andrew D. Johnston, Ryo Maekawa, Fabien Delahaye, Netha Ulahannan, Kami Kim and John M. Greally

METHODS

Theory behind combining P-values

A combined p-value with a Fisher's or Stouffer's Method becomes more significant as the number of combined p-values increase. This behavior is considered a major strength of p-value combination methods [1] as shown in the following simplified scenario. Given two gene promoters, G_1 and G_2, and epigenetic modifications overlapping them with effect significances p_1 and p_2, respectively, such that p_1=(0.01, 0.60) and p_2=(0.06, 0.10, 0.20), we would traditionally conclude that G_1 has a significant effect at α<0.05 and deprioritize G_2. However, using the Fisher's Method combined p-value for both p_1 and p_2, we find that the $p_{1,combined}$=0.04 and $p_{2,combined}$=0.04. Even though p_2 has no significant p-values at α=0.05, it has more borderline significant elements than p_1 and, therefore, should be prioritized. In this manner, the overall study power can be improved by not ignoring evidence that falls above arbitrary thresholds only by chance.

P-value combination methods

Of the available methods used in meta-analyses to combine p-values, the most common methods are implemented in SMITE. Each method has strengths and weaknesses and may be useful depending on the type of combined effect that is most interesting to the user. For an explanation of the implemented methods, we consider combining K p-values, $p_{1\ldots k}$. The generalized form for combining K p-values from independent experiments in a meta-analysis is:

$$T = \sum_{i=1}^{K} w_i H(p_i) \qquad (1)$$

where wi represents weights and H is a transformation of p-values [2].

(1) Stouffer's method [3] first applies the inverse standard normal CDF transformation of each p_i such that:

$$\Phi^{-1}\left(1-\frac{p_i}{2}\right) = Z_i \qquad (2)$$

and then calculates a combined statistic as:

$$Z_{Stouffer} = \frac{\sum_{i=1}^{k} Z_i}{\sqrt{k}} \quad Z_{Stouffer} \sim N(0,1) \qquad (3)$$

Stouffer's method is convenient because one can easily include weights for the individual components $w_{i\ldots k}$ [4] such that:

$$Z_{Stouffer} = \frac{\sum_{i=1}^{k} w_i Z_i^*}{\sqrt{\sum_{i=1}^{k} w_i^2}} \quad Z_{Stouffer} \sim N(0,1) \quad (4)$$

(2) Fisher's method [5] is another straightforward way to combine p-values where:

$$T_{Fisher} = -2\sum_{i=1}^{k} \ln(p_i) \quad T_{Fisher} \sim \chi^2_{2k} \quad (5)$$

Fisher's method is optimal when trying to assess the joint significance between nodes in an interaction network, which is generally a sum between the node scores. There is some evidence that Fisher's method can lose power when there are a few large p-values compared to the rest of the p-values [6].

(3) Sidak's Adjustment [7] is equivalent to taking the most significant effect within a region, which is a common practice in genomics research, but it includes an additional penalty for the number of p-values considered such that:

$$p_{Sidak} = 1-(1-min(p_{1...k}))^k \quad (6)$$

(4) The binomial method is an intuitive approach to combining p-values that relies on finding the probability under a Binomial distribution of finding significant p-values given a series of tests. By defining a threshold, α, and finding the total number of p-values less than or equal to alpha, we calculate the probability under a binomial distribution of finding the result or a more extreme result such that:

$$p_{binomial} \sim Bin(k, \frac{\sum_{i=1}^{k} I(p_i \le \alpha)}{k} \quad (7)$$

Cholesky decomposition for correlated p-values

The existence of the Cholesky decomposition for a correlation matrix is known so that given a symmetric, positive, and definite correlation matrix, Σ_{ij}, there is an upper triangular matrix with positive diagonal entries, C_{ij}, so that Σ_{ij}=$C_{ij}^T C_{ij}$. In the literature, one major use of this decomposition has been to correlate random variables that are independent. One example comes from Hoyland, Kaut, and Wallace [8], where they state that given $\tilde{X}$, an n-dimensional N (0, 1) random variable with a correlation structure indicating mutual independence, then the $\tilde{Y}$ = C$\tilde{X}$ is an n-dimensional N (0, 1) random variable with correlation matrix that depends on C and is not longer mutually independent. By rearranging this equation it is apparent that mutually independent random variables may be achieved by finding the product of inverse of the Cholesky decomposition of a correlation matrix and a N(0,1) random variables that are assumed to be dependent, $C^{-1}\tilde{Y}$ = $\tilde{X}$. Thus, as the inverse standard normal CDF transformation $\Phi^{-1}\left(1-\frac{p_{ijk}}{2}\right) = Z_{ijk}$ results in N(0,1) distributed random variables, when considering correlated p-values we can use ${C_{ij}}^{-1}Z_{ijk} = Z_{ijk}^*$ to find a mutually independent random variables. This use of the Cholesky decomposition has been previously demonstrated [6].

Notation used to explain SMITE algorithm

For the theory behind SMITE, we use {ijk} to denote each p-value associated with an interval and gene, e.g. one p-value in a specific gene's promoter. We use {ij} to denote each interval associated with a particular gene, e.g. a single combined p-value representing all p-values within a specific gene's promoter. We use {.j} to denote each interval as it associates with all genes, e.g. a single weight associated with all promoters. We use {i} to denote each gene. Therefore, we define genes as G_i for i in 1,2…I and genomic intervals as R_{ij} for j in $1,2…J$ related to G_i (*e.g.* a specific gene's promoter and body). Within each R_{ij}, we find the N overlapping p-values, p_{ijk} for k in 1…N_{ij}. Weights $w_{\cdot j}$ are defined for each $R_{\cdot j}$,

Program runtime and requirements

We benchmarked the running time for SMITE on a Windows machine with 4 GB of RAM at approximately 45 minutes, while the running time on a high performance computing Linux cluster was approximately 30 minutes, depending on the number and resolution of loaded modifications. SMITE is a pipeline with multiple steps, so the runtime is not necessarily reflective of the actual analysis time, which would involve more user interaction with the data. Memory intensive processes like the spin-glass algorithm may fail unless the system has enough free RAM, which in our tests required roughly 1.6 GB. On memory-poor systems, we have found that this sometimes requires saving the working data set object, freeing up memory on the system and within R, and loading the object again before running network algorithms in SMITE.

Infection of human foreskin fibroblasts (HFF) with *Toxoplasma gondii (T. gondii)*

To benchmark SMITE, we obtained a large multifaceted genomics dataset from a controlled experiment studying the genomic effects on human foreskin fibroblasts (HFF) following infection by *T. gondii*. This dataset is part of a separate manuscript in preparation, and will be made available as a public resource after manuscript submission. The HFFs were obtained from ATCC CRL-1634 Hs27 LOT:4012886. After being received from ATCC, they were labeled as P16 (Passage 16) and a lab stock was created that was labeled P17. All experiments were done using P17-P20, and HFFs were discarded after P20. HFFs were grown in Dulbecco's modified Eagle medium (DMEM; Gibco) supplemented with 10% fetal bovine serum (FBS; HyClone), 100 U/mL penicillin (Gibco), 100 µg/mL streptomycin (Gibco) and 2 mM L-glutamine (HyClone) and were maintained at 37°C with 5% CO_2. *T. gondii* type I tachyzoites (RH) were repeatedly passaged with HFFs until the host infections were synchronized. Following synchronization of the infection, the tachyzoites were released by passing the infected cells through a 25 gauge needle three times and centrifuging at 3000 rpm for 8 minutes. Next, 75 cm^2 flasks containing confluent HFFs were infected using a multiplicity of infection (MOI) of 3. After 24 hours, the proportion of infected host cells per flask was calculated by identification of parasite rosettes adjacent to the nuclei of the infected host cells using a light microscope, and quantifying the proportion of cells showing this pattern. When flasks were found to have at least 80% infected HFFs, they were harvested by scraping. An uninfected flask containing cells to be used as controls was also harvested in parallel. The harvested cells were centrifuged at 1,300 rpm for 5 minutes. Cells were harvested such that both RNA and DNA could be extracted from the same flask for each biological replicate, and three replicates of uninfected and infected HFFs were harvested in total. We expect that these intracellular parasites could alter multiple host cellular pathways, especially pathways related to infection, inflammation, metabolism and host cell cycle [9, 10].

Genomic DNA was extracted from cells using a protocol developed by the Einstein Epigenomics Facility. The cells were incubated in 10 ml of a DNA extraction buffer (10 mM Tris-HCl (Fisher), 0.1 M EDTA (Sigma-Aldrich), 0.5% SDS (Sigma-Aldrich), and 10 µl of 20 mg/mL RNaseA (NEB)) at 37°C for one hour, then incubated with 50 µl Proteinase K (Life Technologies) at 50°C overnight. Next, 10 ml of saturated phenol (Fisher) was added to the DNA extraction buffer and mixed slowly at room temperature for 15 minutes then centrifuged at 3000 rpm for 10 minutes at room temperature. The supernatant was transferred to a new 50 ml falcon tube, and this process was repeated twice more with saturated phenol. The process was repeated three additional times substituting the phenol with chloroform (Sigma). Subsequently, the sample was pipetted into a dialysis bag (Fisher) and put sequentially into three 500 ml baths of 0.2x saline-sodium citrate buffer (Fisher). Finally, the dialysis bags were placed on PEG crystals (Sigma) allowing the water to be removed by osmosis. The DNA was collected from the dialysis bag and stored at 4°C for further analysis (see HELP-tagging and HELP-GT). Gene expression (directional RNA-seq), DNA methylation (HELP-tagging [11]), and DNA hydroxymethylation (HELP-GT [12]) profiles were generated from the three uninfected and three *T. gondii*-infected HFF samples, using additional information about *cis*-regulatory element locations from ChIP-seq annotations of histone modifications of IMR90 human fibroblasts. Through simultaneous alignment of RNA-seq reads to a combined hg19-*Toxoplasma* genome, we find that the relative proportions of parasite and host were similar between replicates (**Figure S1**). Although the RH strain of *T. gondii* was used in our experiments, we chose to align the reads to the ME49 v9.0 *Toxoplasma* reference genome, as it was more completely annotated at the time of alignment, was shown to be 97.6% identical to the RH strain [13], and is the most common strain infecting humans.

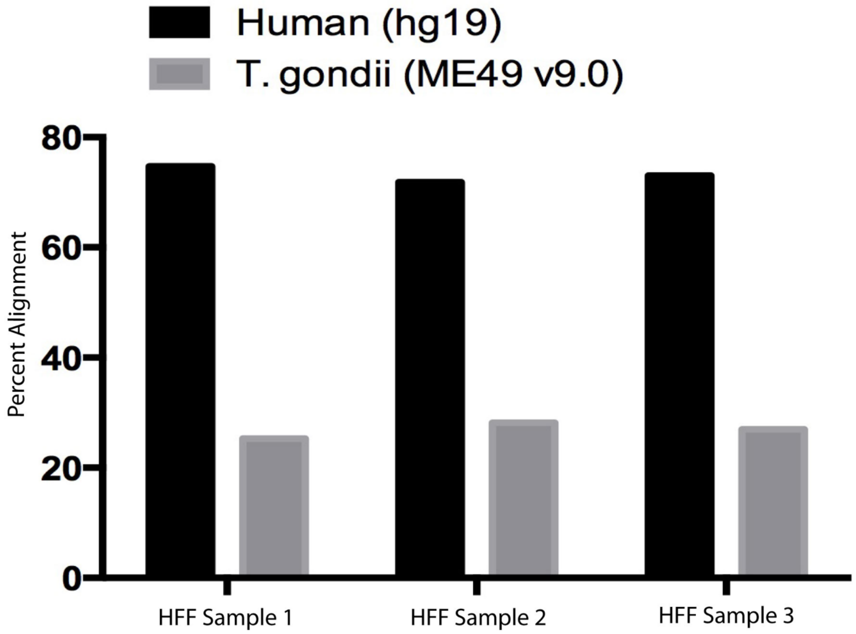

Figure S1: For each HFF + *T. gondii* replicate we aligned the RNA-seq reads to a composite hg19-*Toxoplasma* genome. We assess the proportion of reads that aligned to each genome separately to demonstrate consistency between and within samples.

HELP-tagging and HELP-GT

The HELP-tagging assay was developed by our group [11] and is a high-throughput approach to assay DNA methylation genome-wide. Samples were treated with the restriction enzymes HpaII or MspI, both of which recognize a CCGG motif but have a differential ability to digest DNA depending on the presence of 5-methylcystosine (5-mc). After preparing and sequencing libraries from digested DNA, the relative number of sequencing tags between the HpaII and MspI channels indicated the relative DNA methylation at a specific locus. Using HELP-tagging, we were able to assay DNA methylation at approximately 2 million loci. We used a modification of HELP-tagging, HELP-GT, to assay genome-wide DNA hydroxymethylation [12]. HELP-tagging does not discriminate between methylated and hydroxymethylated CpG dinucleotides, but the use of the bacteriophage T4 beta-glucosyltransferase (BGT) can catalyze the addition of a glucose moiety to the hydroxyl group of 5-hydroxymethlcytosines (5-hmC), which interferes with the ability of MspI to digest DNA at these CpG dinucleotides. A third comparison after BGT treatment followed by MspI treatment allows for the detection of 5-hmC levels within each sample. Both HELP-tagging and HELP-GT were performed on the control and infected *T. gondii* samples. The resulting significance is obtained from two-sided T-testing with 4 degrees of freedom.

RNA-seq

Directional RNA-seq was performed on the uninfected and *T. gondii*-infected samples. Total RNA was extracted from each of the samples using a Trizol protocol. Cells were pelleted and resuspended in ice-cold PBS (Fisher). Following this, 1 ml of the Trizol reagent (Life Technologies) was added, with 5 minutes of incubation at room temperature and centrifugation to remove cell debris. The supernatant was transferred to new tube and 0.2 ml of chloroform (Sigma) was added and the tube was centrifuged. The aqueous phase was transferred and added to 0.5 ml of isopropyl alcohol. The samples were incubated at room temperature for 10 minutes and centrifuged. The RNA pellet was washed with 75% ethanol, air-dried, and resuspended in nuclease-free water. The extracted RNA was then depleted for ribosomal RNA using the Ribo-zero kit (Epicentre Biotechnologies). After sequencing the mRNA-enriched library, HTSeq was used to calculate the total counts at each human gene, and the Bioconductor package DESeq was used to compare relative expression between uninfected and *T. gondii* infected samples using negative binomial testing. For the purposes of this study, we examined only the human host gene expression and epigenome, querying for changes induced in the host by *T. gondii* infection.

Defining *cis*-regulatory elements in human fibroblasts

The histone modification profiles of a human fibroblastic cell type, IMR-90, assayed by the ENCODE project [14] were obtained. We used the H3K4me1, H3K27ac, and H3K27me3 modifications to define *cis*-regulatory elements in this cell type. We processed the data using an adaptation of an imaging signal processing algorithm to define the locations of chromatin constituents with minimal data transformation [15, 16]. We defined genomic context relative to the gene transcription start site (TSS) by the criteria defined in **Table S1**.

**Genomic Context**	**Criteria**
RefSeq Gene Promoter	± 2 kb from TSS
RefSeq Gene Body	Gene Body TSS to TES<br>(minus promoter region)
Active Enhancer	H3K4me1+, H3K27ac+
Poised Enhancer	H3K4me1+, H3K27me3+

Table S1: To analyze the *T. gondii* HFF dataset, genomic contexts were defined using the RefSeq gene annotation track from the UCSC genome browser and IMR90 histone mark combinations available from ENCODE. TSS: transcription start site.

Incorporating *a priori* hypotheses into SMITE analysis

SMITE incorporates three different points within its pipeline where the user can alter the final outcome dramatically depending on *a priori* information. First, users can include a priori information about how modifications within a specific genomic context should be weighted. Distance from the TSS is a popular method to weight epigenetic effects, given that epigenetic states may reflect transcription factor (TF) binding, and TFs are often assumed to maintain a relationship with a gene within a specific distance from the gene's TSS [17]. Alternatively, as is the case in other network analysis tools like Epimods [18], a researcher may only focus on the most significant p-value within a particular genomic context, although SMITE allows the use of the Sidak correction that accounts for multiple comparisons. Second, users can control *a priori* information about the relationship between transcription and a modification given a particular genomic context. Though the relationship between transcription and epigenetic modifications can be complicated, some relationships have been studied extensively and are generally found to be true like DNA methylation in gene promoters being associated with transcriptional silencing [19], whereas DNA methylation in a gene body is associated with increased expression [20–22]. By giving the user has the option to define a relationship for each modification-genomic context pairing, SMITE results in functional modules enriched for more easily interpretable gene effects. Third, when scoring nodes, a linear combination of weights can be provided to address *a priori* research goals defining the relative importance of a component score toward the final scores and functional modules (e.g., functional modules enriched for relating transcription and enhancer modifications).

**Annotation**	**Level**	**Relationship with increasing expression**	**SMITE: Full weight**	**SMITE: Reduced weight**
Expression	Gene	NA	0.1	0.5
DNA Methylation	Gene promoters	Decreasing	0.04	0.5
	Gene Bodies	Increasing	0.01	0.0001
	Active Enhancers	Bidirectional	0.2	0.0001
	Poised Enhancers	Bidirectional	0.2	0.0001
DNA Hydroxymethylation	Gene promoters	Bidirectional	0.04	0.0001
	Gene Bodies	Bidirectional	0.01	0.0001
	Active Enhancers	Bidirectional	0.2	0.0001
	Poised Enhancers	Bidirectional	0.2	0.0001

Table S2. To score the *T. gondii* HFF dataset, the relationship between each modification and gene expression can be indicated in a genomic context-specific manner. Here, DNA methylation at gene promoters will have an inverse relationship with expression, and DNA methylation at gene bodies will have a positive relationship with gene expression. All other effects will be maximized regardless of their direction. In addition, the weighting vectors indicate that the modules we detect in the SMITE-Full model will be driven by gene expression, DNA methylation and DNA hydroxymethylation at enhancers, and they were chosen so that the sum of the weights would be 1. The modules that we detect in the SMITE-Reduced model will be driven by gene expression and promoter DNA methylation.

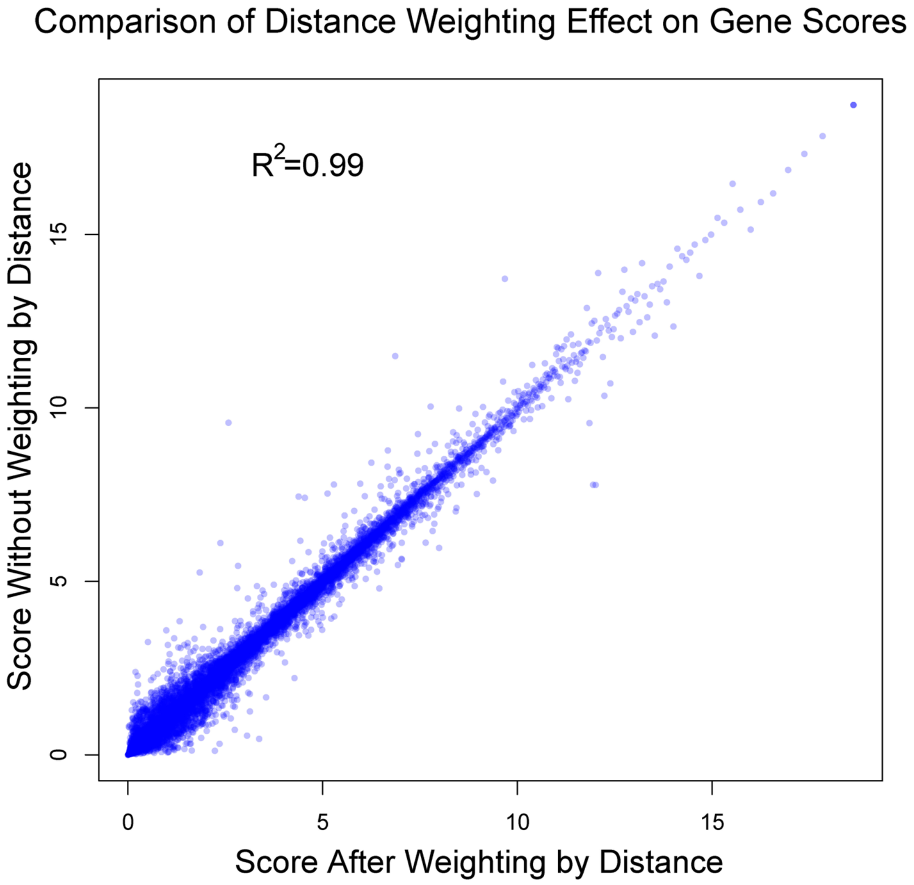

Figure S2: To explore the effect of using altered weights, w_{ijk}, we requested that no weights be used and compared it to weighting by distance. From the overall scores derived from p-values and the R^2=0.99 it is apparent that the overall scores are very concordant and the effect of the weight choice is minimal.

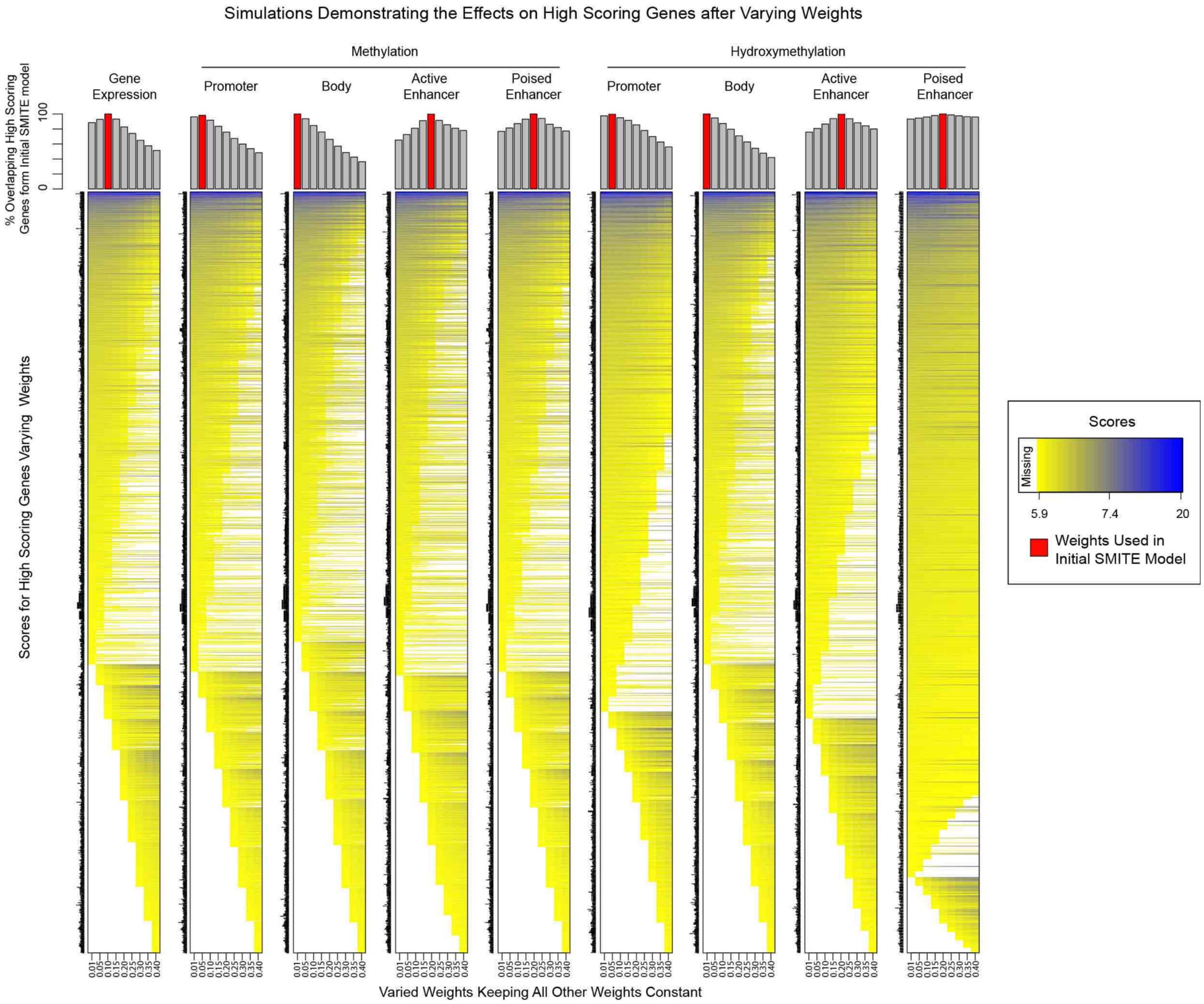

Figure S3: To explore the effect of using altered weights, $w_{\cdot j}$, we fixed all weights except for one which was varied between 0.01, a value that down weights the component severely, and 0.4, a value that makes the component the dominant one in the model. Then for each iteration, we requested the highest scoring genes (genes that were above the random null score distribution) and showed their scores. Finally, we show for each iteration the proportion of high scoring genes under the original model (weights shown in red) that remain. For each component, there is a subset of genes that remains high scoring despite the choice in weight, and a subset of genes that emerges as more weight is placed on the component.

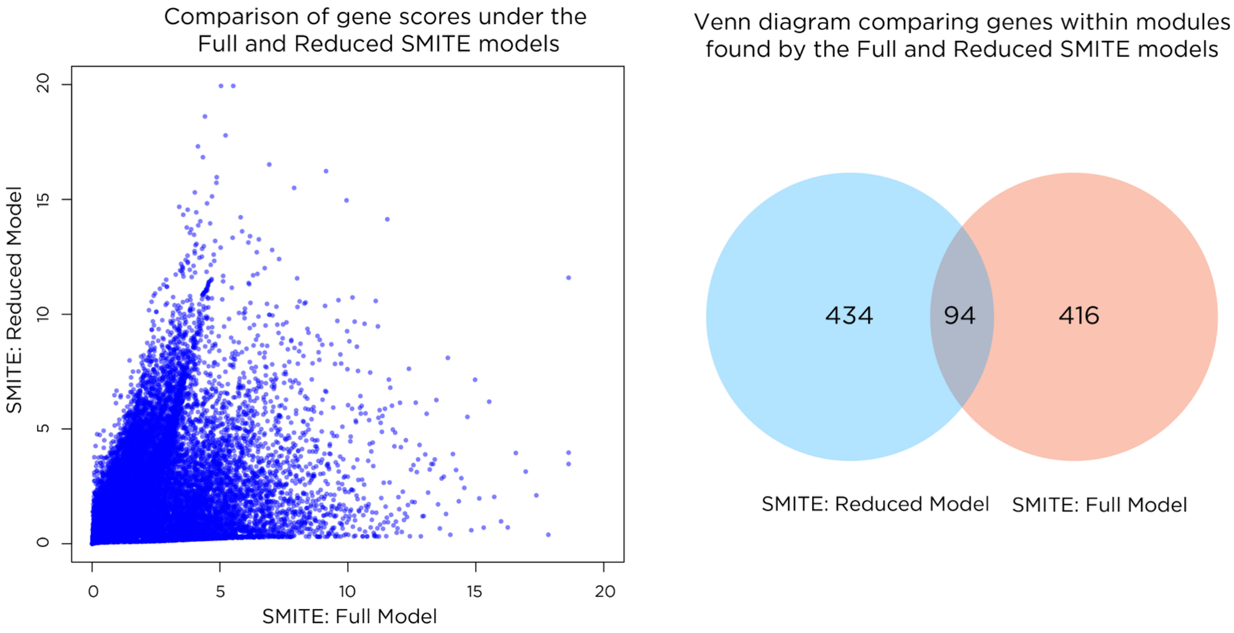

Figure S4: Under the reduced SMITE model (SMITE-R) and the full SMITE model (SMITE-F) we compare the overall scores of genes to show how gene scores vary greatly under these two very different model choices. The Venn diagram shows that there is very little overlap in the downstream high scoring genes.

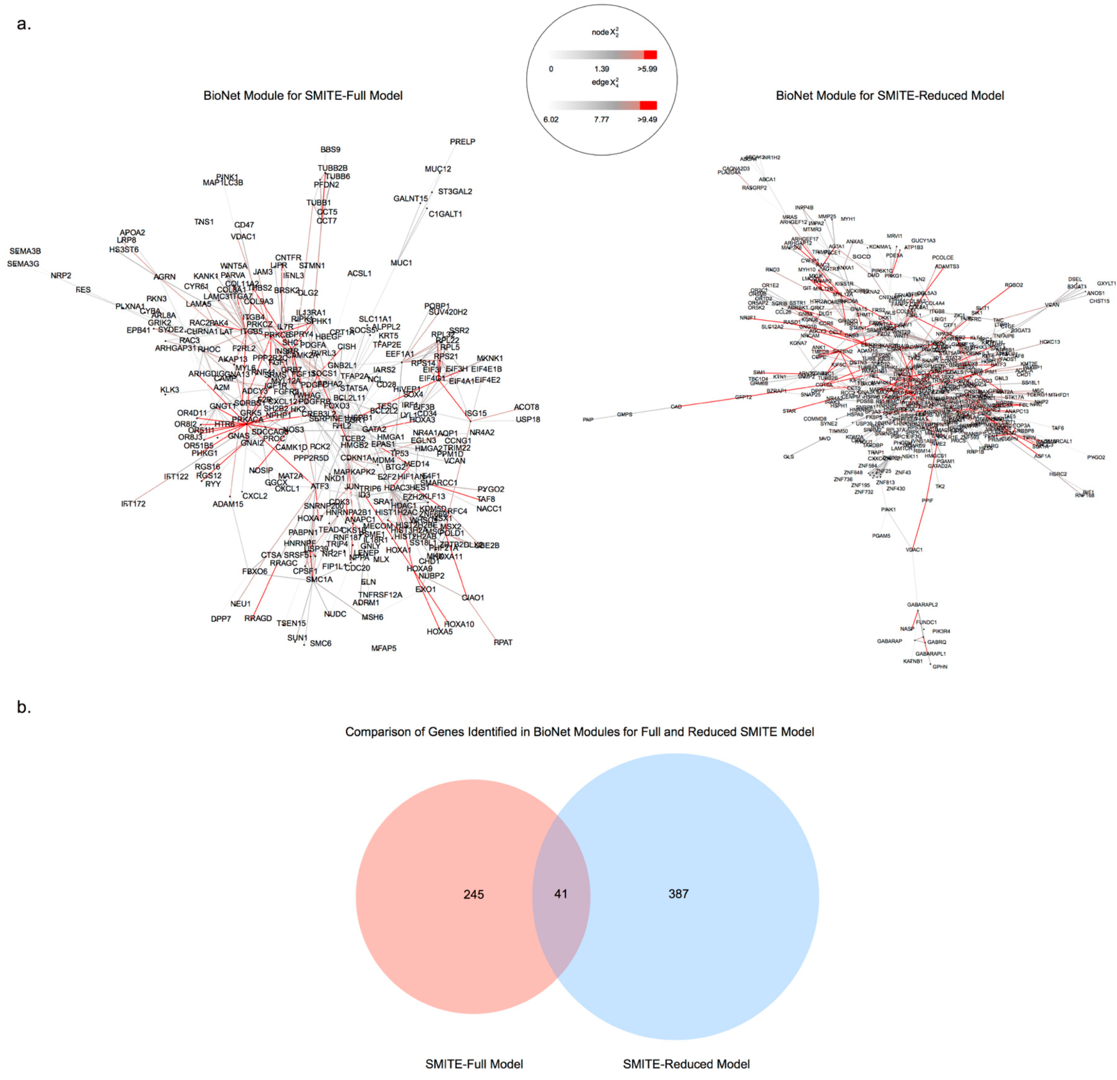

Figure S5. Using the reduced SMITE model (SMITE-R) and the full SMITE model (SMITE-F), we requested the BioNet module using the heinz algorithm. (a) The detected modules are shown, and within them there are hotspots that roughly correspond to the modules detected by the spin-glass algorithm. (b) From a Euler diagram of the module genes, it is clear that there is very little overlap between the identified genes between SMITE-R and SMITE-F.

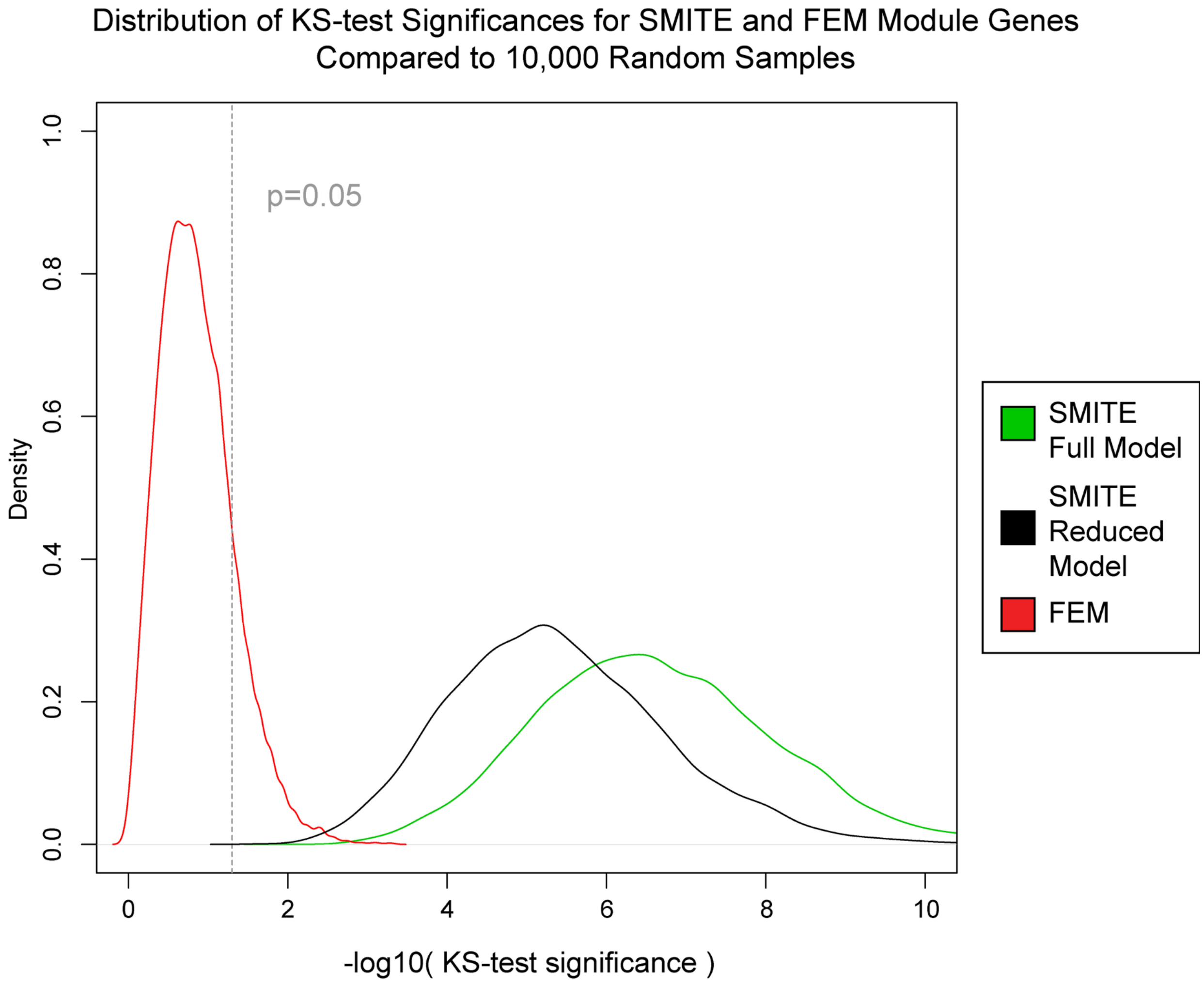

Figure S6: Because a KS-test between SMITE module genes (roughly 500) and SMITE scored genes (roughly 23,000) will generally be significant when compared with a KS-test between FEM module genes (roughly 200) and FEM scored genes (roughly 6,000), we instead used a sampling approach to determine the KS-test significance. For FEM, the reduced SMITE model (SMITE-R), and the full SMITE model (SMITE-F), we computed 10,000 random samples of equal size to the identified modules and plot the significance. On average, FEM module genes are not statistically different when compared to all FEM genes; whereas, SMITE-R and SMITE-F achieve a greater statistical significance.

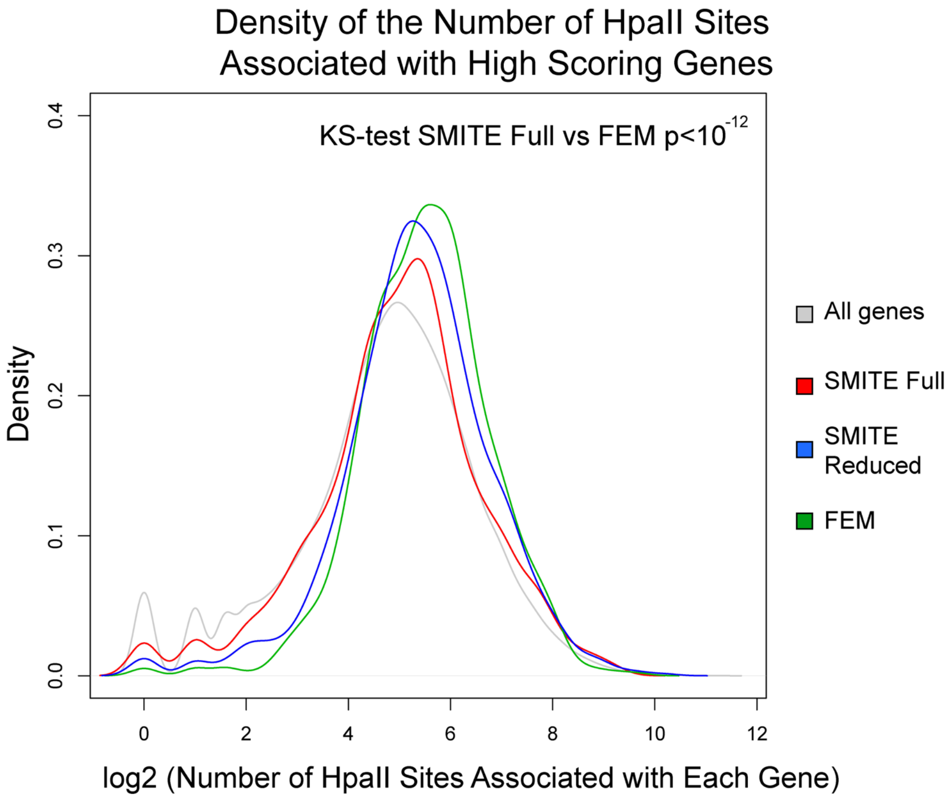

Figure S7: We found the number of HpaII sites that were assayed for DNA methylation and DNA hydroxymethylation, and thus the number of p-values, associated with each gene, and we looked for the distribution of the number of p-values associated with high scoring genes for all genes, SMITE-F genes, SMITE-R genes, and FEM genes. We find that the SMITE-F model outperforms the FEM model in terms of bias toward genes associated with more p-values, having a statistically significant different distribution (KS-test p-value $<10^{-12}$).

Appendix 1. R code for analyzing *T. gondii* HFF dataset with SMITE

```
options(stringsAsFactors=FALSE)

library(SMITE)

library(data.table)

#load modification p-values and curate data

methylation <- as.data.frame(fread("SMITE_Meth.txt", header=TRUE, stringsAsFactors
=FALSE))
methylation <- methylation[-which(is.na(methylation[,4])),]
methylation[, 4] <- replace(methylation[, 4], methylation[, 4] == 0, min(subset(methylation[, 4],
methylation[, 4] !=0 ), na.rm=TRUE))
methylation<-methylation[,c(1:3,5,4)]

hydroxyl <- as.data.frame(fread("SMITE_Hydroxy.txt",header=TRUE,stringsAsFactors =F))
hydroxyl < -hydroxy[-which(is.na(hydroxy[,4])),]
hydroxyl[, 4] <- replace(hydroxyl[,4], hydroxyl[, 4] == 0, min(subset(hydroxyl[, 4], hydroxyl[, 4] !=
0), na.rm=TRUE))
hydroxyl <- hydroxyl[,c(1:3,5,4)]

#load gene expression p values
genes <- read.table("SMITE_Exp.txt", header=TRUE, stringsAsFactors =FALSE)

genes <- genes[-which(is.na(genes[, 3])), ]
genes <- genes[-which(duplicated(genes[, 1])), ]
genes[, 1] <- convertGeneIds(gene_IDs=genes[, 1], ID_type ="ensemble", ID_convert_to
="symbol")
genes <- genes[-which(is.na(genes[, 1])), ]
genes <- split(genes, genes[, 1])
genes <- lapply(genes, function(i){
        if(nrow(as.data.frame(i)) > 1){
                I <- i[which(i[, 3] == min(i[, 3],na.rm=TRUE))[1], ]
                }
        return(i)})
genes <- do.call(rbind, genes)
genes <- genes[, -1]
genes[, 2] <- replace(genes[, 2], genes[,2] == 0, min(subset(genes[, 2], genes[, 2] != 0),
na.rm=TRUE))
genes[grep("-Inf", genes[, 1]), 1] <- (-1)
genes[grep("Inf", genes[, 1]), 1] <- 1
expression <- genes
colnames(expression) <- c("effect","pval")


#Create annotation with gene symbols and enhancers

data(hg19_genes_bed)
activeenhancers<-read.table("ActiveEnhancer.bed", header=F, stringsAsFactors =F)
poisedenhancers<-read.table("PoisedEnhancer.bed", header=F, stringsAsFactors =F)
```

```
Toxo_annotation <- makePvalueAnnotation(data=hg19_genes,
otherdata=list(active_enhancers=activeenhancers, poised_enhancers=poisedenhancers),
gene_name_col=5, other_tss_distance=20000)

#fill in expression data

Toxo_annotation <- annotateExpression(Toxo_annotation, expression, effect_col=1,
pval_col=2)

#fill in modification data

Toxo_annotation <- annotateModification(Toxo_annotation, methylation,
weight_by_method="Stouffer", weight_by=c(promoter="distance", body="distance",
active_enhancers="distance", poised_enhancers="distance"), verbose=TRUE,
mod_corr=TRUE)

Toxo_annotation <- annotateModification(Toxo_annotation, hydroxyl,
weight_by_method="Stouffer", weight_by=c(promoter="distance", body="distance",
active_enhancers="distance", poised_enhancers="distance"), verbose=TRUE,
mod_type="hydroxymeth", mod_corr=TRUE)

#create a pvalue object that will count the effect of the h3k4me1 as bidirectional

 Toxo_annotation <- makePvalueObject(Toxo_annotation,
effect_directions=c(methylation_promoter="decrease", methylation_body="increase",
methylation_active_enhancers="bidirectional", methylation_poised_enhancers="bidirectional",
hydroxymeth_promoter="bidirectional", hydroxymeth_body="bidirectional",
hydroxymeth_active_enhancers="bidirectional",
hydroxymeth_poised_enhancers="bidirectional"))

#normalize the pvalues compared to expression

Toxo_annotation <- normalizePval(Toxo_annotation, ref="expression", method="rescale")


#score with all four features contributing

Toxo_annotation <- scorePval(Toxo_annotation, weights=c(expression=.1,
methylation_promoter=0.04, methylation_body=0.01, methylation_active_enhancers=0.2,
methylation_poised_enhancers=0.2, hydroxymeth_promoter=0.04, hydroxymeth_body=0.01,
hydroxymeth_active_enhancers=.2, hydroxymeth_poised_enhancers=.2))

#load REACTOME

load(system.file("data", "Reactome.Symbol.Igraph.rda", package="SMITE"))

 #run Spin-glass using REACTOME network

Toxo_annotation <- runSpinglass(Toxo_annotation, REACTOME, maxsize=100,
num_iterations=1000, simplify=TRUE)

#run goseq on individual modules to determine bias
```

```
Toxo_annotation <- runGOseq(Toxo_annotation, coverage=read.table(system.file("extdata", "hg19_symbol_hpaii.sites.inbodyand2kbupstream.bed", package="SMITE"), stringsAsFactors = F), type="kegg")

#search go seq output for keywords

searchGOseq(Toxo_annotation, "Mapk")

#Draw a network

plotModule (Toxo_annotation, which.network=11, layout="fr")
```

Appendix 2. R code for analyzing *T. gondii* HFF dataset with FEM

```
#load FEM and dependencies

library("igraph")
library("marray")
library("corrplot")
library("graph")
library("AnnotationDBI")
library(FEM)

#load a converting package because FEM requires Entrez IDs
sym2eg<-AnnotationDbi::as.list(org.Hs.eg.db::org.Hs.egSYMBOL2EG)

#Using a combination of bedTools and R we assembled for DNA methylation:

#1) average of all effects within 200 bp from a gene TSS
#2) if no effects were found, the average of effects over the first exon
#3) if no effects were found, taking the average over 1,500 bp around the TSS

#Read methylation data into R and merge it to make statM

statM.1<-read.table("Epimods_Meth.genesoverlapping200bp.txt")
statM.2<-read.table("Epimods_Meth.genesoverlapping1stExon.txt")
statM.3<-read.table("Epimods_Meth.genesoverlapping1500bp.txt")

# find average effects for 400bp region flanking gene tss

statM.1<-statM.1[,c(5,10,11)]
temp<-split(statM.1, statM.1[,1])
temp<-lapply(temp, function(x){
if(nrow(x)>1){
x[1,2]<-mean(x[,2],na.rm=T)
x[1,3]<-min(x[,3],na.rm=T)
x<-x[1,]}
x})

statM.1<-as.matrix(do.call(rbind, temp))
if(any(is.na(statM.1[,2]))){statM.1<-statM.1[-which(is.na(statM.1[,2])),]}

# find average effects for first exon

statM.2<-statM.2[which(!statM.2[,5]%in%statM.1[,1]),]
statM.2<-statM.2[,c(5,10,11)]

temp<-split(statM.2, statM.2[,1])
temp<-lapply(temp, function(x){
if(nrow(x)>1){
x[1,2]<-mean(x[,2],na.rm=T)
x[1,3]<-min(x[,3],na.rm=T)
x<-x[1,]
```

```
}
x})

statM.2<-as.matrix(do.call(rbind, temp))

if(any(is.na(statM.2[,2]))){statM.2<-statM.2[-which(is.na(statM.2[,2])),]}

# find average effects for 3000bp region flanking gene tss

statM.3<-statM.3[which(!statM.3[,5]%in%statM.2[,1]),]

statM.3<-statM.3[,c(5,10,11)]
temp<-split(statM.3, statM.3[,1])
temp<-lapply(temp, function(x){

if(nrow(x)>1){
x[1,2]<-mean(x[,2],na.rm=T)
x[1,3]<-min(x[,3],na.rm=T)
x<-x[1,]}
x})

statM.3<-as.matrix(do.call(rbind, temp))
if(any(is.na(statM.3[,3]))){statM.3<-statM.3[-which(is.na(statM.3[,2])),]}
statM<-rbind(statM.1,statM.2,statM.3)

#convert names to entrez

M.entrez<-sapply(statM[,1], function(i){return(sym2eg[[i]])})
M.entrez<-lapply(M.entrez, function(i){if(length(i)>1){i<-i[1]}; if(length(i)==0){i<-NA};i})
M.entrez<-do.call(c, M.entrez)
statM[,1]<-M.entrez
statM<-statM[-which(is.na(statM[,1])),]
rownames(statM)<-statM[,1]
statM<-statM[,-1]
statM<-as.data.frame(statM)

#Read expression data into R to make statR
statR<-read.table("Epimods_Exp.txt")

#convert gene names

M.entrez<-sapply(statR[,1], function(i){return(sym2eg[[i]])})
M.entrez<-lapply(M.entrez, function(i){if(length(i)>1){i<-i[1]}; if(length(i)==0){i<-NA};i})
M.entrez<-do.call(c, M.entrez)
statR[,1]<-M.entrez
statR<-statR[-which(is.na(statR[,1])),]
statR<-statR[-which(is.na(statR[,2])),]

#for overlapping entrez genes take the minimum p value and the average t stat

temp<-split(statR, statR[,1])
temp<-lapply(temp, function(x){
if(nrow(x)>1){
x[1,2]<-mean(x[,2],na.rm=T)
```

```
x[1,3]<-min(x[,3],na.rm=T)
x<-x[1,]}
x})

statR<-as.matrix(do.call(rbind, temp)[,-1])

#Load graph

load("Reactome.Symbol.Igraph.rda")
REACTOME.df<-get.data.frame(REACTOME)
M.entrez<-sapply(REACTOME.df[,1], function(i){return(sym2eg[[i]])})
M.entrez<-lapply(M.entrez, function(i){if(length(i)>1){i<-i[1]}; if(length(i)==0){i<-NA};i})
M.entrez<-do.call(c, M.entrez)
 REACTOME.df[,1]<-M.entrez

M.entrez<-sapply(REACTOME.df[,2], function(i){return(sym2eg[[i]])})
M.entrez<-lapply(M.entrez, function(i){if(length(i)>1){i<-i[1]}; if(length(i)==0){i<-NA};i})
M.entrez<-do.call(c, M.entrez)

 REACTOME.df[,2]<-M.entrez
REACTOME.df<-REACTOME.df[-which(duplicated(REACTOME.df)),]
REACTOME.df<-REACTOME.df[-which(is.na(REACTOME.df[,1])),]
 REACTOME.df<-REACTOME.df[-which(is.na(REACTOME.df[,2])),]
REACTOME<-graph.data.frame(REACTOME.df, directed=F)
REACTOME<-induced_subgraph(REACTOME,V(REACTOME)[igraph::degree(REACTOME) >
0])
REACTOME<-igraph::simplify(REACTOME)
cl <- clusters(REACTOME)
delete.clusters <- which(cl$csize < 100)
vertices.to.remove <- which(cl$membership %in% delete.clusters)
REACTOME <- delete.vertices(REACTOME, vertices.to.remove)

#Find common genes between all 3 and subset

if(any(is.na(statR[,1]))){statR<-statR[-which(is.na(statR[,1])),]}
if(any(is.na(statM[,1]))){statM<-statM[-which(is.na(statM[,1])),]}
graph_genes<-V(REACTOME)$name
meth_genes<-rownames(statM)
exp_genes<-rownames(statR)
common_genes<-intersect(intersect(meth_genes, exp_genes), graph_genes)

#subset datasets
statR<-statR[which(rownames(statR)%in%common_genes),]
statM<-statM[which(rownames(statM)%in%common_genes),]
REACTOME<-induced_subgraph(REACTOME, common_genes)
REACTOME<-
delete.vertices(REACTOME,subset(V(REACTOME)$name,igraph::degree(REACTOME)==0))
statM<-t(sapply(V(REACTOME)$name, function(i){statM[which(rownames(statM)==i),]}))
statR<-t(sapply(V(REACTOME)$name, function(i){statR[which(rownames(statR)==i),]}))

cl <- clusters(REACTOME)
delete.clusters <- which(cl$csize < 100)
vertices.to.remove <- which(cl$membership %in% delete.clusters)
```

```
REACTOME <- delete.vertices(REACTOME, vertices.to.remove)

graph_genes<-V(REACTOME)$name
meth_genes<-rownames(statM)
exp_genes<-rownames(statR)
common_genes<-intersect(intersect(meth_genes, exp_genes), graph_genes)

statM<-statM[which(rownames(statM)%in%common_genes),]
statR<-statR[which(rownames(statR)%in%common_genes),]
REACTOME<-induced_subgraph(REACTOME, common_genes)

 statM<-as.data.frame(statM)
 statM[,1]<-as.numeric(statM[,1])
 statM[,2]<-as.numeric(statM[,2])
 statM<-as.matrix(statM)

statR<-as.data.frame(statR)
 statR[,1]<-as.numeric(statR[,1])
 statR[,2]<-as.numeric(statR[,2])
 statR<-as.matrix(statR)

#create input

input<-list(as.matrix(statM), as.matrix(statR), get.adjacency(REACTOME))



#run FEM

output<-DoFEMbi(statM.m=input[[1]], statR.m=input[[2]], adj.m=input[[3]],sizeR.v=c(8,100))
```

REFERENCES

1. Rosenthal R: **Combining results of independent studies**. *Psychol Bull* 1978, **85**.

2. Zaykin DV: **Optimally weighted Z-test is a powerful method for combining probabilities in meta-analysis.** *J Evol Biol* 2011, **24**:1836–1841.

3. Stouffer S, DeVinney LN, Suchman E: *The American Soldier, Adjustment During Army Life*. Princeton, US: Princeton University Press; 1949.

4. Lipták T: **On the combination of independent tests.** *Magyar Tud Akad Mat Kutato Int Közl* 1958, **3**:171–197.

5. Fisher RA: *Statistical Methods for Research Workers*. Fourth edition. Edinburgh: Oliver and Boyd; 1932.

6. Zaykin DV, Zhivotovsky LA, Westfall PH, Weir BS: **Truncated product method for combining P-values.** *Genet Epidemiol* 2002, **22**:170–185.

7. Sidak Z: **Rectangular confidence regions for the means of multivariate normal distributions**. *J Am Stat Assoc* 1967, **62**:626–633.

8. Høyland, Kaut K, Wallace M, W S: **A Heuristic for Moment-Matching Scenario Generation**. *Computational Optimization and Applications* 2003.

9. Aliberti J: **Host persistence: exploitation of anti-inflammatory pathways by Toxoplasma gondii.** *Nat Rev Immunol* 2005, **5**:162–170.

10. Blader IJ, Saeij JP: **Communication between Toxoplasma gondii and its host: impact on parasite growth, development, immune evasion, and virulence.** *APMIS* 2009, **117**:458–476.

11. Suzuki M, Jing Q, Lia D, Pascual M, McLellan A, Greally JM: **Optimized design and data analysis of tag-based cytosine methylation assays.** *Genome Biol* 2010, **11**:R36.

12. Bhattacharyya S, Yu Y, Suzuki M, Campbell N, Mazdo J, Vasanthakumar A, Bhagat TD, Nischal S, Christopeit M, Parekh S, Steidl U, Godley L, Maitra A, Greally JM, Verma A: **Genome-wide hydroxymethylation tested using the HELP-GT assay shows redistribution in cancer.** *Nucleic Acids Res* 2013, **41**:e157.

13. Lau Y-L, Lee W-C, Gudimella R, Zhang G, Ching X-T, Razali R, Aziz F, Anwar A, Fong M-Y: **Deciphering the Draft Genome of Toxoplasma gondii RH Strain.** *PLoS ONE* 2016, **11**:e0157901.

14. ENCODE Project Consortium: **An integrated encyclopedia of DNA elements in the human genome.** *Nature* 2012, **489**:57–74.

15. Wijetunga NA, Delahaye F, Zhao YM, Golden A, Mar JC, Einstein FH, Greally JM: **The meta-epigenomic structure of purified human stem cell populations is defined at cis-**

regulatory sequences. *Nat Commun* 2014, **5**:5195.

16. Delahaye F, Wijetunga NA, Heo HJ, Tozour JN, Zhao YM, Greally JM, Einstein FH: **Sexual dimorphism in epigenomic responses of stem cells to extreme fetal growth.** *Nat Commun* 2014, **5**:5187.

17. Sikora-Wohlfeld W, Ackermann M, Christodoulou EG, Singaravelu K, Beyer A: **Assessing computational methods for transcription factor target gene identification based on ChIP-seq data.** *PLoS Comput Biol* 2013, **9**:e1003342.

18. West J, Beck S, Wang X, Teschendorff AE: **An integrative network algorithm identifies age-associated differential methylation interactome hotspots targeting stem-cell differentiation pathways.** *Sci Rep* 2013, **3**:1630.

19. Deaton AM, Bird A: **CpG islands and the regulation of transcription.** *Genes Dev* 2011, **25**:1010–1022.

20. Hellman A, Chess A: **Gene body-specific methylation on the active X chromosome.** *Science* 2007, **315**:1141–1143.

21. Ball MP, Li JB, Gao Y, Lee JH, LeProust EM, Park IH, Xie B, Daley GQ, Church GM: **Targeted and genome-scale strategies reveal gene-body methylation signatures in human cells.** *Nat Biotechnol* 2009, **27**:361–368.

22. Suzuki M, Oda M, Ramos MP, Pascual M, Lau K, Stasiek E, Agyiri F, Thompson RF, Glass JL, Jing Q, Sandstrom R, Fazzari MJ, Hansen RS, Stamatoyannopoulos JA, McLellan AS, Greally JM: **Late-replicating heterochromatin is characterized by decreased cytosine methylation in the human genome.** *Genome Res* 2011, **21**:1833–1840.
